# Allogeneic stem cell transplantation in fully MHC-matched Mauritian cynomolgus macaques recapitulates diverse human clinical outcomes

**DOI:** 10.1038/s41467-017-01631-z

**Published:** 2017-11-10

**Authors:** Benjamin J. Burwitz, Helen L. Wu, Shaheed Abdulhaqq, Christine Shriver-Munsch, Tonya Swanson, Alfred W. Legasse, Katherine B. Hammond, Stephanie L. Junell, Jason S. Reed, Benjamin N. Bimber, Justin M. Greene, Gabriela M. Webb, Mina Northrup, Wolfram Laub, Paul Kievit, Rhonda MacAllister, Michael K. Axthelm, Rebecca Ducore, Anne Lewis, Lois M. A. Colgin, Theodore Hobbs, Lauren D. Martin, Betsy Ferguson, Charles R. Thomas Jr., Angela Panoskaltsis-Mortari, Gabrielle Meyers, Jeffrey J. Stanton, Richard T. Maziarz, Jonah B. Sacha

**Affiliations:** 1 Vaccine and Gene Therapy Institute, Oregon Health & Science University, 505 NW 185th Avenue, Beaverton, OR 97006 USA; 2 Oregon National Primate Research Center, Oregon Health & Science University, 505 NW 185th Avenue, Beaverton, OR 97006 USA; 3 Division of Medical Physics, Department of Radiation Medicine, Oregon Health & Science University, 3181 SW Sam Jackson Park Road, Portland, OR 97239 USA; 4 Division of Blood and Marrow Transplantation, Department of Pediatrics, University of Minnesota, 2450 Riverside Avenue, Minneapolis, MN 55454 USA; 5 Division of Hematology and Medical Oncology, Knight Cancer Institute, Oregon Health & Science University, 3181 SW Sam Jackson Park Road, Portland, OR 97239 USA

## Abstract

Allogeneic hematopoietic stem cell transplantation (HSCT) is a critically important therapy for hematological malignancies, inborn errors of metabolism, and immunodeficiency disorders, yet complications such as graft-vs.-host disease (GvHD) limit survival. Development of anti-GvHD therapies that do not adversely affect susceptibility to infection or graft-vs.-tumor immunity are hampered by the lack of a physiologically relevant, preclinical model of allogeneic HSCT. Here we show a spectrum of diverse clinical HSCT outcomes including primary and secondary graft failure, lethal GvHD, and stable, disease-free full donor engraftment using reduced intensity conditioning and mobilized peripheral blood HSCT in unrelated, fully MHC-matched Mauritian-origin cynomolgus macaques. Anti-GvHD prophylaxis of tacrolimus, post-transplant cyclophosphamide, and CD28 blockade induces multi-lineage, full donor chimerism and recipient-specific tolerance while maintaining pathogen-specific immunity. These results establish a new preclinical allogeneic HSCT model for evaluation of GvHD prophylaxis and next-generation HSCT-mediated therapies for solid organ tolerance, cure of non-malignant hematological disease, and HIV reservoir clearance.

## Abstract (web summary)

Rhesus macaques are not ideal for studying response to allogeneic hematopoietic stem cell transplant (allo-HSCT) owing to complex MHC genetics that prevent full MHC-matching. Here the authors show that inbred Mauritian-origin cynomolgus macaques are a superior preclinical model of allogeneic stem cell transplantation that mimics diverse clinical outcomes of human allo-HSCT.

## Introduction

Although allogeneic hematopoietic stem cell transplantation (HSCT) is a foundational treatment capable of functionally curing malignant and nonmalignant hematological pathologies, severe complications such as graft rejection, opportunistic infection, organ failure, and graft-vs.-host disease (GvHD) are frequent. Despite high-resolution MHC-matching and aggressive immunosuppressive regimens, GvHD is the leading cause of post-allogeneic HSCT non-relapse morbidity and mortality^1^. Approximately 60% of allogeneic HSCT recipients are estimated to develop GvHD, with survival rates as low as 5% in patients with advanced grade steroid-refractory acute GvHD^2, 3^. Therefore, new immunomodulatory modalities are needed that selectively limit GvHD while retaining infectious disease immunity and the protective graft-vs.-tumor effect of allogeneic HSCT.

The development of GvHD treatments requires a reproducible and accurate animal model capable of recapitulating human physiology and pathology. Large animal HSCT models utilizing dogs, pigs, or sheep are limited by the availability of species-specific reagents such as immunophenotyping antibodies. Murine GvHD models have provided important mechanistic insight into the development and pathology of the GvHD immune response, yet direct clinical translation of findings from the mouse model is often hindered by species-specific differences between murine and human immune systems, homogenous genetics of inbred mouse strains, divergent anatomical sources of human and murine donor cells, and the dysregulated microbiome of mice raised in pathogen-free housing^4, 5^. By contrast, given their close phylogenetic proximity to humans, nonhuman primates (NHP), such as rhesus and cynomolgus macaques, provide physiologically relevant models for a range of degenerative, genetic, age-associated, and infectious human diseases^6–8^. Indeed, immunotherapeutics can directly cross-react with both the human and macaque targets, allowing rapid translation of new laboratory discoveries into clinical trials^9–11^.

Rhesus macaques are an outbred NHP population with remarkably complex MHC genetics. Each animal expresses up to twenty MHC-I molecules^12^, thereby precluding their use as a preclinical model of fully MHC-matched allogeneic HSCT. By contrast, Mauritian-origin cynomolgus macaques (MCM), an insular population that underwent a population bottleneck ~400 years ago, have only seven fully characterized MHC haplotypes^13, 14^. Here we show that fully MHC-matched MCMs can be used for an ideal preclinical model of allogeneic HSCT, mirroring a range of clinical outcomes including graft rejection, lethal GvHD, CMV reactivation, and recipient-specific tolerance during full donor T cell chimerism. These results establish a new, physiologically relevant model of fully MHC-matched allogeneic HSCT for addressing critical challenges in HSCT treatment, such as the development of novel GvHD prophylactic regimens to disentangle graft-vs.-host and graft-vs.-tumor immunity, and provide a platform for testing experimental HSCT applications, such as HSCT-mediated clearance of the latent HIV reservoir.

## Results

### Engraftment failure in Mauritian cynomolgus macaques

**Fig. 1:**
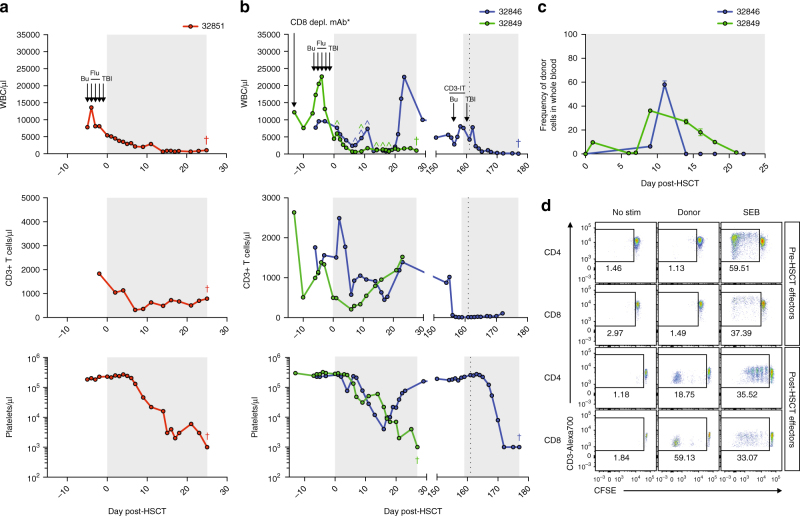
Primary and secondary engraftment failure in MHC-matched MCM post-allogeneic HSCT. **a**, **b** Longitudinal white blood cell (WBC), T cell, and platelet absolute counts in recipient macaques 32851 (**a**), 32846 (**b**), and 32849 (**b**). Immune conditioning regimens are indicated in top panels (Bu = busulfan, Flu = fludarabine, TBI = total body irradiation, CD3-IT = CD3-immunotoxin). CD8 depleting mAb was administered only to 32849, indicated by an asterisk (*). Colored circumflexes (^) indicate time points at which donor-derived cells were detected in whole blood by chimerism assays. Crosses (†) indicate time of necropsy. Normal WBC reference range = 5,600–184,000/μl; normal platelet reference range = 134,000–564,000/μl. Shaded gray boxes in **a**–**c** indicate tacrolimus treatment period. Vertical dotted line in **b** indicates second transplant of 32846. **c** Longitudinal donor chimerism levels in 32846 and 32849 as measured by Illumina sequencing whole blood genomic DNA across SNPs differing between donor and recipient. Graphs display mean ± SEM frequencies of donor-derived cells as measured by sequencing two (32849) or three (32846) SNPs. **d** Mixed lymphocyte reactions assessing levels of CD4+ and CD8+ T cell alloreactivity in recipient macaque 32846 pre-HSCT and 28 days post-HSCT. Plots display proliferation of CFSE-labeled recipient T cells in response to irradiated donor cells, as measured by frequency of CFSE-lo cells. Plots are gated on live, CD3+ singlets, and either CD4+ or CD8+ cells. Recipient cells cultured alone (no stim) or with Staphylococcus enterotoxin B (SEB) served as negative and positive controls, respectively.

Due to the complex immunogenetics of rhesus macaques^12^, previous NHP allogeneic HSCT experiments have primarily utilized single MHC haplotype-matched donor-recipient pairs, which invariably results in either host-mediated graft rejection or donor-mediated acute lethal GvHD^6, 10, 15^. Even HSCT between fully MHC-matched sibling rhesus macaques generated only transient mixed donor chimerism with minimal T cell engraftment ending with either graft rejection or lethal infectious disease complications^16, 17^. To overcome the limitation posed by rhesus macaque MHC genetic complexity, we set out to establish an allogeneic HSCT model based on MCM, a geographically isolated NHP population with highly limited immunogenetic diversity^13, 14^. Indeed, unrelated fully MHC-matched donor-recipient MCM pairs for allogeneic HSCT could readily be identified (Table 1). To ensure the clinical relevance of the model, we adopted protocols utilized in patients at the Oregon Health & Science University Bone Marrow Transplant Department including a reduced intensity conditioning (RIC) regimen of busulfan, fludarabine, and low-dose total body irradiation (TBI), peripheral blood stem cell mobilization of donors, and GvHD prophylaxis of tacrolimus plus methotrexate^18, 19^ (Supplementary Figs. 1 and 2). Following RIC and fully MHC-matched HSCT, the first recipient, animal 32851, experienced primary engraftment failure characterized by extended leukopenia, anemia, and thrombocytopenia eventually necessitating euthanasia (Fig. 1a). No donor chimerism was detected in whole blood as measured by a DNA surveyor nuclease assay targeting a single-nucleotide polymorphism (SNP) differing between donor and recipient (Supplementary Fig. 3). This primary engraftment failure was likely a consequence of the sub-optimal CD34+ stem cell dose (Table 1). Leukapheresis procedures on NHP weighing <10 kg remains a technical challenge, precluding the routine use of peripheral blood mobilized stem cells in NHP experiments. To overcome this obstacle, we optimized the leukapheresis collection parameters specifically for donor MCM as small as 3.5 kg and performed multiple procedures per donor, resulting in the ability to consistently achieve more optimal CD34+ stem cell doses for all subsequent HSCT (Supplementary Fig. 2; Table 1).

**Table 1:** Summary of hematopoietic stem cell transplants in MHC-matched Mauritian cynomolgus macaques

Recipient			Donor						
ID	MHC haplotypes	Sex	ID	MHC haplotypes	Sex	Total cells transplanted	CD34 dose/kg	CD3 dose/kg	Outcome
32851	M1/M1	M	32847	M1/M1	M	2.20 × 10^9^	1.55 × 10^6^	N.D.	Primary engraftment failure
32846	M2/M3	M	32843	M2/M3	M	8.50 × 10^9^	5.26 × 10^6^	3.74 × 10^8^	Secondary engraftment failure
32849	M3/M3	M	32843	M3/M3	M	4.90 × 10^9^	9.42 × 10^6^	2.80 × 10^8^	Secondary engraftment failure
32846*	M2/M3	M	32843	M2/M3	M	1.49 × 10^9^	4.43 × 10^6^	6.40 × 10^7^	Primary engraftment failure
33450	M1/M3	F	33452	M1/M3	M	1.05 × 10^10^	3.33 × 10^7^	4.05 × 10^8^	Lethal GvHD
33455	M1/M3	M	33452	M1/M3	M	6.27 × 10^9^	1.06 × 10^7^	3.73 × 10^8^	Lethal GvHD
33454	M2/M4	F	33461	M2/M4	M	4.80 × 10^8^	7.80 × 10^6^	5.18 × 10^7^	Stable Chimerism
34666	M2/M6	F	33456	M2/M6	F	1.60 × 10^9^	8.84 × 10^6^	1.06 × 10^8^	Stable Chimerism

N.D. not determined

Table summarizes the eight HSCTs performed in this study, listed in chronological order. Asterisk (*) indicates the second transplant of recipient macaque 32846

The next two recipients, 32846 and 32849, underwent the same RIC regimen described above, but received HSCT containing higher CD34+ stem cell doses (Table 1; Supplementary Fig. 1). Donor chimerism was initially detected in the whole blood of both recipients, as measured by the DNA surveyor nuclease assay, but in both recipients the donor signal was rapidly lost despite immunosuppression with tacrolimus and methotrexate (Fig. 1b; Supplementary Fig. 3). In 32846, the appearance of whole blood donor chimerism was accompanied by a rebound in the white blood cell (WBC) and neutrophil counts at day 11 post-transplant, but donor signal disappeared along with a sharp decline in WBC at day 14, indicating graft rejection. Indeed, the WBC and platelets rebounding at day 21 were recipient-derived. In contrast, the transient donor chimerism in 32849 was not accompanied by a change in WBC and the recipient immune system did not rebound, resulting in euthanasia. Because the DNA surveyor assay is only qualitative, we also utilized quantitative deep sequencing across SNPs, which confirmed the transient donor chimerism in blood measured by the DNA surveyor assay (Fig. 1c; Supplementary Fig. 3). Mixed lymphocyte reactions (MLR) from 32846 revealed induction of potent donor-specific T cell immunity post-HSCT, suggesting active host T cell immune-mediated rejection of the graft (Fig. 1d). Based on these results, and the observation that fludarabine incompletely depletes CD3+ T cells in MCM (Fig. 1a, b), we replaced fludarabine in the conditioning regimen with a CD3-specific immunotoxin (CD3-IT) previously shown in NHP to mimic the lymphocyte depleting activities of anti-thymocyte globulin in humans^20^. Despite the ability of CD3-IT to potently reduce CD3 counts in NHP, the variability of NHP CD3 sequences has precluded wide usage^21^. However, every MCM examined to date expresses the highly susceptible CD3 sequence, suggesting CD3-IT will be universally effective in MCM (Supplementary Fig. 4). We utilized this new conditioning regimen to perform a second HSCT in 32846 using the same donor (Table 1; Supplementary Fig. 1). Despite significant depletion of peripheral CD3+ T cells by CD3-IT, we observed no donor chimerism and the graft was again rejected, likely due to strong anti-donor immunity in residual, tissue-resident recipient T cells (Fig. 1b, d). Thus, sufficient minor histocompatibility antigens exist between fully MHC-matched MCMs to lead to immune-mediated rejection of allogeneic cells.

### Development of lethal GvHD post-HSCT in MCM

**Fig. 2:**
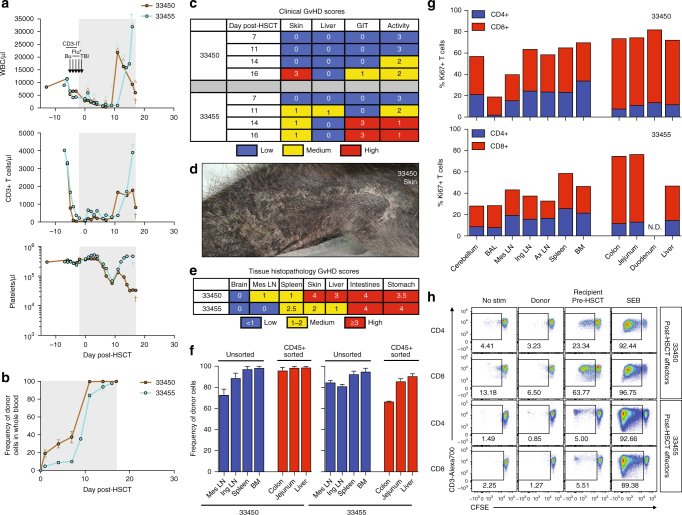
Lethal GvHD in fully MHC-matched MCM post-allogeneic HSCT. **a** Longitudinal white blood cell (WBC), T cell, and platelet absolute counts in recipient macaques 33450 and 33455. Immune conditioning regimens are indicated in top panels (Bu = busulfan, Flu = fludarabine, TBI = total body irradiation, CD3-IT = CD3-immunotoxin). *Fludarabine was administered only to 33450. Colored circumflexes (^) indicate timepoints at which donor-derived cells were detected in whole blood by chimerism assays. Crosses (†) indicate time of necropsy. Normal WBC reference range = 5,600–18,4000/μl; normal platelet reference range = 134,000–564,000/μl. Shaded gray boxes in **a**, **b** indicate tacrolimus treatment period. **b** Longitudinal donor chimerism levels as measured by Illumina sequencing whole blood genomic DNA across SNPs differing between donor and recipient. Graphs display mean ± SEM frequencies of donor-derived cells as measured by two SNPs. **c** Longitudinal clinical GvHD scoring. Scoring criteria described in Methods section. **d** Multifocally extensive exfoliative dermatitis of thoracic limb of recipient macaque 33450. **e** Blinded tissue histopathology GvHD scoring at time of necropsy. Scoring criteria described in Methods section. **f** Tissue donor chimerism levels at time of necropsy as measured by Illumina sequencing genomic DNA across SNPs differing between donor and recipient. Graphs display mean ± SEM frequencies of donor-derived cells as measured by sequencing two SNPs. To eliminate non-immune cell contamination, colon, jejunum, and liver cell preparations were sorted for CD45+ cells prior to genomic DNA extraction. **g** Frequencies of Ki67+ T cells in tissues at time of necropsy, as determined by intracellular Ki67 flow cytometric staining. **h** Mixed lymphocyte reactions assessing levels of CD4+ and CD8+ T cell alloreactivity in recipient macaques 33450 and 33455 post-HSCT (at time of necropsy). Plots display proliferation of CFSE-labeled recipient T cells in response to irradiated donor or pre-HSCT recipient cells, as measured by frequency of CFSE-lo cells. Plots are gated on live, CD3+ singlets, and either CD4+ or CD8+ cells. Recipient cells cultured alone (no stim) or with SEB served as negative and positive controls, respectively.

Given the ability of CD3-IT to effectively deplete recipient T cells, we next explored if the improved CD3-IT-containing RIC regimen might facilitate full engraftment in MCM lacking donor-specific immunity. Two HSCT-naive MCMs, 33450 and 33455, received the CD3-IT, busulfan, and low-dose TBI RIC regimen followed by fully MHC-matched HSCT (Table 1; Supplementary Fig. 1). In contrast to previous transplants, whole blood donor chimerism was present as early as day 1 post-HSCT and detected at every subsequent time point (Fig. 2a). Both recipients experienced a massive expansion of WBC and CD3+ T cell counts, and concomitantly reached full donor chimerism in whole blood (Fig. 2a, b). At day 12 post-HSCT, 33450 was reported for extensive hand rubbing and itchy palms, a clinical harbinger of GvHD. Indeed, despite immunosuppression with methotrexate and tacrolimus (Supplementary Fig. 5), both animals progressed rapidly with clinical symptoms of GvHD involving the skin, liver, and gastrointestinal tract necessitating euthanasia (Fig. 2c; Supplementary Fig. 6). Most striking was the emergence of erythematous rash and exfoliative dermatitis, which upon microscopic analysis exhibited the GvHD hallmarks of vacuolar interface dermatitis and necrotic keratinocytes (Fig. 2d; Supplementary Fig. 7). Importantly, these changes were observed in hair follicles, excluding interface drug eruption as a potential cause. Both animals also developed anorexia, weight loss, and diarrhea, indicating gastrointestinal GvHD. Indeed, histologic features of GvHD were present throughout gastrointestinal tract tissues, including partial to complete destruction of crypts characterized by high numbers of apoptotic crypt epithelial cells and near-complete loss of intestinal villi (Supplementary Fig. 7). Independent, blinded quantification of the histopathological changes confirmed extensive GvHD in skin, liver, and gastrointestinal tract, mirroring the clinical symptoms observed (Fig. 2e). Given the high level of GvHD pathology, we next measured the level of donor T cell chimerism and T cell activation within affected organs such as the liver, colon, and jejunum, as well as in primary and secondary lymphoid organs. Consistent with the GvHD-mediated destruction of recipient tissue, the majority of T cells within affected organs and lymphoid tissues were donor-derived (Fig. 2f). Furthermore, although both CD4+ and CD8+ T cells exhibited markers of active proliferation including Ki67, this activated cell phenotype represented the majority of CD8+ T cells, particularly within GvHD-affected organs (Fig. 2g; Supplementary Fig. 8). In vitro MLR performed at the time of euthanasia confirmed high-level donor chimerism with anti-recipient alloreactivity as robust T cell proliferation occurred in response to pre-HSCT recipient cells, but not donor cells (Fig. 2h). These results demonstrate that fully MHC-matched MCM can experience significant post-HSCT GvHD, which is not controlled by standard human tacrolimus and methotrexate prophylaxis schedules, indicating the need for more stringent prophylactic GvHD regimens.

### Generation of stable chimeras with full donor engraftment

**Fig. 3:**
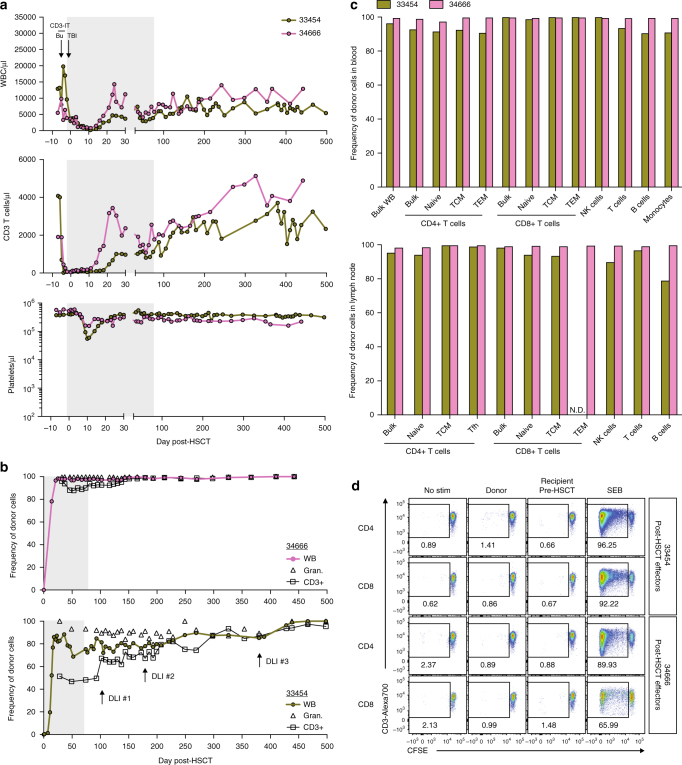
Stable, multi-lineage donor chimerism in MHC-matched MCM post-allogeneic HSCT. **a** Longitudinal white blood cell (WBC), T cell, and platelet absolute counts in recipient macaques 33454 and 34666. Immune conditioning regimens are indicated in top panels (Bu = busulfan, TBI = total body irradiation, CD3-IT = CD3-immunotoxin). Normal WBC reference range = 5,600–184,000/μl; normal platelet reference range = 134,000–564,000/μl. Shaded gray boxes in **a**, **b** indicate tacrolimus treatment period. **b**, **c** Donor chimerism levels as measured by Illumina sequencing genomic DNA across SNPs differing between donor and recipient. Specific populations were isolated by flow cytometric cell sorting of ACK-treated whole blood prior to genomic DNA extraction. Longitudinal whole blood (WB), granulocyte, and T cell donor chimerism levels shown in **b**. Timepoints of donor lymphocyte infusions (DLIs) into 33454 are indicated with arrows in **b** (bottom panel); DLI #1 = 1 × 10^7^ CD3+ cells/kg, DLI #2 = 5 × 10^7^ CD3+ cells/kg, DLI #3 = 5 × 10^7^ CD3+ cells/kg. Comprehensive immune cell subset donor chimerism levels in whole blood (top) and lymph node (bottom) shown in **c**, measured 428 days (33454) or 193 days (34666) post-HSCT. N.D. = not determined due to insufficient cell numbers. **d** Mixed lymphocyte reactions assessing levels of CD4+ and CD8+ T cell alloreactivity in recipient macaques 33454 and 34666 post-HSCT (354 and 298 days post-HSCT, respectively). Plots display proliferation of CFSE-labeled recipient T cells in response to irradiated donor or pre-HSCT recipient cells, as measured by frequency of CFSE-lo cells. Plots are gated on live, CD3+ singlets, and either CD4+ or CD8+ cells. Recipient cells cultured alone (no stim) or with SEB served as negative and positive controls, respectively.

Post-transplant cyclophosphamide administration and T cell co-stimulation blockade are promising GvHD preventative approaches capable of blunting the development of alloreactive T cells following HSCT^22–24^. Given the lethal GvHD observed in MCM recipients treated with tacrolimus and methotrexate, we intensified our GvHD prophylaxis regimen by replacing methotrexate with single high-dose post-transplant cyclophosphamide and the CD28 costimulation-blockade reagent Belatacept (Supplementary Fig. 1). Two recipients, 33454 and 34666, underwent busulfan, CD3-IT, and low-dose TBI RIC coupled with antibiotic-mediated gut decontamination followed by fully MHC-matched HSCT (Table 1). We detected whole blood donor chimerism in 33454 and 34666 by DNA surveyor assay beginning at days 11 and 14 post-HSCT, respectively, which preceded a rise in WBC, neutrophil, and CD3+ T cell counts (Fig. 3a; Supplementary Fig. 9). Neither animal developed anemia or thrombocytopenia of sufficient severity to require a blood transfusion, indicating the RIC and GvHD regimen was well tolerated. Remarkably, no clinical symptoms of GvHD accompanied the rapid donor engraftment, and both animals were successfully weaned off immunosuppression by day 80 post-HSCT (Fig. 3a; Supplementary Figs. 10 and 11). Indeed, both recipients remain clinically stable over 1 year post-HSCT without immunosuppression and show no clinical, serum chemistry, or hematologic evidence of GvHD.

Quantitative assessment of donor chimerism in peripheral blood granulocytes and CD3+ T cells revealed that 34666 was 100% donor chimeric in both cellular compartments by day 32 post-HSCT, although a transient decrease in T cell chimerism occurred before again returning to full donor T cell chimerism at day 151 post-HSCT (Fig. 3b). Full donor engraftment in both granulocytes and T cells was subsequently maintained throughout the remainder of the study. In contrast, 33454 initially achieved only mixed chimerism, with 100% granulocyte chimerism and 51% T cell donor chimerism in peripheral blood at day 28 post-HSCT. Because recipients with mixed donor chimerism are at increased risk of graft rejection and malignant disease relapse, donor lymphocyte infusions (DLI) are commonly performed to achieve 100% donor chimerism^25^. Therefore, we performed a series of DLIs with escalating doses in 33454. Although an immediate increase in T cell chimerism followed the first DLI, a total of three DLI procedures spread over nearly 1 year were required to reach full T cell donor chimerism (Fig. 3b; Supplementary Fig. 12). The DLI procedures were well tolerated and we observed no clinical signs of GvHD due to the infusion of donor lymphocytes in the absence of immune suppression. The extent of donor chimerism across immune cell subsets in peripheral blood and lymphoid tissue mirrored that of the whole blood values with 100% of all immune cells in 34666 being of donor origin in both anatomical locations while 33454 exhibited split donor chimerism with 100% CD8+ T cell chimerism, yet only 80% donor chimerism in the B-cell compartment in lymph node (Fig. 3c).

Both post-transplant cyclophosphamide and CD28 co-stimulation blockade can eliminate the development of alloreactive T cells and induce T cell anergy^10, 23^. Given the durability and extent of donor engraftment without GvHD, we performed in vitro MLR to assess recipient-specific alloreactive cellular immune responses. In both 33454 and 34666, the engrafted T cells exhibited complete tolerance to recipient antigens (Fig. 3d). However, this immune tolerance towards recipient cells was not simply the result of a defective immune system. Following HSCT, CMV reactivation occurred in both recipients, one of which was treated with short-term valganciclovir. CMV viremia was subsequently controlled to undetectable levels in both recipients in the absence of treatment, and we detected strong CMV-specific immune responses (Supplementary Fig. 13). In particular, 33454 developed anti-CMV T cell responses directed against epitopes not recognized pre-HSCT or in the stem cell donor. Therefore, post-transplant cyclophosphamide and CD28 costimulation blockade GvHD prophylaxis is able to induce recipient-specific tolerance while maintaining infectious disease immunity.

## Discussion

Together, these data demonstrate that fully MHC-matched MCM represent an ideal model of allogeneic HSCT, recapitulating many clinical outcomes of RIC allogeneic HSCT such as primary and secondary graft failure, GvHD, mixed donor chimerism with responsiveness to DLI, CMV reactivation and subsequent control, and durable full donor multi-lineage chimerism. The establishment of this robust, pre-clinical model will facilitate experiments across a range of regenerative medicine applications to help translate new therapies into the clinic. Our studies have revealed conditions that generate rapid onset graft rejection and GvHD between fully MHC-matched MCM undergoing allogeneic HSCT, which can be exploited for subsequent studies. Overall, this minor antigen alloreactivity mirrors the immune response in patients receiving HSCT grafts from MHC-matched unrelated and related donors and thus augurs well for the clinical relevance of this model. Indeed, a direct extension of the research presented here is the exploration of novel GvHD prophylaxis regimens such as the post-transplant cyclophosphamide, belatacept, and tacrolimus regimen that induced full donor chimerism and recipient-specific tolerance in fully MHC-matched MCM. Experimental approaches to limit GvHD through modulation of the T cell content in stem cell grafts such as depletion of naive cells can also be tested^26^. Further, because many human reagents cross-react with macaque targets, novel investigative GvHD prophylaxis therapeutics can be directly tested. For example, the Tim-3/Gal-9 and TIGIT negative checkpoint receptor pathways are implicated in GvHD severity, and we have previously shown macaque cross-reactivity for human-specific reagents targeting these pathways^27–30^.

The limited, defined MHC genetics of MCM allows for straightforward selection of donor-recipient pairs exhibiting any degree of MHC matching desired without the need for specialized macaque breeding programs. This is especially advantageous for modeling HSCT-induced tolerance across MHC barriers for solid organ transplantation. In addition to the ability to select fully MHC-matched donor-recipient pairs as demonstrated here, MHC haplotype-mismatched MCM can be used to investigate approaches such as HLA-haploidentical HSCT treatments being proposed for patients with aplastic or sickle cell anemia^31^. Finally, induced pluripotent stem cells (iPSCs) are a promising therapeutic tool for regeneration of damaged tissue and the first clinical iPSC transplants were recently completed successfully^32, 33^. However, rejection of fully MHC-matched allogeneic iPSCs has been documented, and avoiding rejection is a major clinical goal as banks of human iPSCs are currently being established for use in allogeneic transplants^34^. The model described here facilitates testing strategies to avoid rejection of transplanted allogeneic iPSC without inducing tumors in the recipient.

The use of fully MHC-matched MCMs for allogeneic HSCT also represents a powerful new tool for identifying mechanisms leading to HIV reservoir clearance following HSCT. To date there exists only one documented case of functional HIV cure, which occurred following an allogeneic HSCT from a *CCR5* ^*Δ32/Δ32*^ donor^35^. Subsequent case studies of HIV+ patients receiving allogeneic HSCT have led to disappointing results. One patient underwent myeloablative conditioning and received a graft from a *CCR5* ^*Δ32/Δ32*^ donor, but instead of durable HIV remission post-HSCT, a CXCR4-tropic HIV emerged and replicated to high levels resulting in the patient’s death^36^. In two patients who underwent RIC and received HSCT from *CCR5* ^*+/+*^ donors, cell-associated HIV DNA could not be found post-HSCT and both patients initially experienced antiretroviral therapy (ART)-free HIV remission, yet eventually relapsed with HIV viremia^37, 38^. Inherent difficulties in studying HIV cure in patients undergoing HSCT for cancer such as lack of standardization of ART, immune conditioning, HSCT source and dose, immune suppression regimens, ethical considerations of ART interruption, and limitations on tissue sampling of patients hamper research into the mechanisms involved in purging the latent reservoir. Therefore, the allogeneic HSCT NHP model described here will accelerate HIV cure research by facilitating a rigorous and controlled investigation of the mechanisms of post-HSCT HIV reservoir clearance. In summary, a new NHP model of fully MHC-matched allogeneic HSCT now exists to address clinically relevant questions in transplantation, infectious disease, and beyond for the betterment of human treatment options.

## Methods

### Experimental animals

A total of nine male and four female MCMs (*Macaca fascicularis)*, 4–9 years of age, housed at the ONPRC were utilized for studies under the approval of the Oregon Health and Science University (OHSU) Institutional Animal Care and Use Committee (IACUC). All macaques in this study were managed according to the ONPRC animal care program, which is fully accredited by AAALAC International and is based on the laws, regulations, and guidelines set forth by the United States Department of Agriculture (e.g., the Animal Welfare Act and its regulations, and the Animal Care Policy Manual), Institute for Laboratory Animal Research (e.g., Guide for the Care and Use of Laboratory Animals, 8th edition), and the Public Health Service Policy on Humane Care and Use of Laboratory Animals.

### CD3 and MHC typing

Genomic DNA was isolated from 200 μl of whole blood following the Qiagen DNeasy protocol (Qiagen, cat. no. 69,504) and eluted in 50 μl buffer AE. CD3ε was amplified in a 25 μl reaction using Phusion Hot Start Flex (New England Biosciences, cat. no. M0536S), 0.4 μM primers designed to amplify exon V (5′-ATTTGGGTGGTTCTTTTGTCACTAATTTGC and 5′-GAATGCCCTTTTGAATGGTCCTCCCTAAAG), and 25 ng genomic DNA. The PCR conditions were 98 °C 00:30/35 cycles: 98 °C 00:10, 55 °C 00:20, 72 °C 00:30/72 °C 5:00 (used for all molecular work unless stated otherwise). The 370-bp amplicons were PCR purified using the Nucleospin Gel and PCR cleanup (Macherey-Nagel, cat. no 740,609.250), Sanger sequenced using the same primers, and analyzed in Geneious.

For determining the susceptibility of MCM to the FN18-based CD3-IT, PBMC from 22 MCMs were stained with the following two panels: CD4-APC, CD8-PB, and Live/Dead Yellow Fixable stain (Invitrogen), and either CD3ε-FITC clone FN-18, or CD3ε-FITC clone SP34. Samples were washed once with FACS buffer (PBS with 10% FCS), fixed with 2% paraformaldehyde, and collected on a Becton Dickinson (BD) LSRII instrument. Analysis was performed using Flow Jo (Tree Star, Inc.), gating progressively on lymphocytes, singlets, live cells, and CD3+ cells.

For MHC typing, genomic DNA was isolated as described above. Small amplicons from exon 2 of MHC class I and MHC class II alleles were amplified in 25 μl reactions using Phusion Hot Start Flex, 0.4 µM primers (MHC I 5′-GCTACGTGGACGACACG and 5′-TCGCTCTGGTTGTAGTAGC, MHC II 5′-CGCTTCGACAGCGAC and 5′-ACTCGCCGCTGCA), and 25–75 ng of genomic DNA (same PCR conditions as above). The 191 and 169-bp amplicons for class I and class II, respectively, were gel purified from a 2% agarose gel using the Nucleospin Gel and PCR cleanup and quantified by NanoDrop 2000 (ThermoFisher Scientific). In preparation for deep sequencing, amplicons from each animal were pooled separately with the class I and class II products pooled at a 4:1 ratio, with 200–350 ng DNA per reaction in a final volume of 16 μl dH_2_O. Samples were barcoded with the NEXTflex Rapid DNA Sequencing Bundle kit and 6 nt barcodes 1–48 (BIOO Scientific, cat. no. 5144–03) (AMPure XP beads, Beckman Coulter, cat. no. A63881) following the protocol with minor modifications: all reagents were used at half volume, and the final PCR (conditions in step C1) was six cycles. Barcoded amplicons were quantified with the Qubit dsDNA high-sensitivity kit (Thermo cat. no. Q32854) on a Qubit 2.0 fluorometer and pooled to 2 nM. The samples were run on an Illumina Miseq using 2 × 250 paired end reads. Illumina sequence data were processed using a custom analysis pipeline written by B.N.B. This pipeline has been made available through DISCVR-Seq (https://github.com/bbimber/discvr-seq), as a module for LabKey Server, an open-source platform for the management of scientific data. Briefly, raw reads were trimmed by sequence quality using Trimmomatic and aligned against a reference library comprised of published MHC sequences using the aligner MOSAIK, version 2.2^39–41^. Perfect matches between reads and reference alleles were scored using custom software that utilized HTS-JDK (http://samtools.github.io/htsjdk/).

### Peri-transplantation conditioning and care

The initial Flu-Bu-TBI reduced intensity conditioning regimen consisted of fludarabine (Flu, 30 mg/m^2^ IV on day −4 through day −2, 90 mg/m^2^ total, manufactured by Ben Venue Laboratories, Bedford, OH, USA), busulfan (Bu, 4–6 mg/kg IV on day −5, manufactured by Otsuka America Pharmaceutical, Rockville, MD, USA), and total body irradiation (TBI, 2–4 Gy delivered at 15–20 cGy/min on day −1 using an Elektra Synergy System). However, as fludarabine did not induce lymphopenia in MCM, it was subsequently replaced by CD3-immunotoxin (0.025 mg/kg BID IV on day −6 through day −3, acquired through the NHP Reagent Resource). Details of the conditioning regimen specific to each recipient are shown in Supplementary Fig. 1. Peri-transplant care consisted of Cefazolin 25 mg/kg IV BID beginning at day −1 until approximately day +30. Enrofloxacin 10 mg/kg IV SID was administered when the absolute neutrophil count (ANC) was <1000 cells/μl whole blood and continued until ANC remained above 1000 cells/μl blood for 48 h. Trimethoprim-sulfamethoxazole 30 mg/kg PO SID to BID was administered from approximately day +10 to day +60. Leukoreduced whole blood or packed red blood cells were transfused when HGB was <6 g/dl or HCT was <20%. Platelet-rich plasma was transfused when platelet counts were <20,000 cells/μl blood or if signs of coagulopathy were observed on physical examination. All blood product transfusions were matched according to the ABO blood group antigen system. Some recipients received G-CSF (Filgrastim or Pegfilgrastim) to enhance donor engraftment. Two recipients (33454 and 34666) received oral antibiotics (1 × 10^6^ U polymyxin B and 500 mg neomycin sulfate) SID beginning day −6 through day +10 to decontaminate the gastrointestinal tract. Details of the peri-transplant care specific to each recipient are shown in Supplementary Fig. 1. Anti-GvHD prophylaxis consisted of tacrolimus, initially administered as a continuous IV drip and subsequently given as an intramuscular (IM) injection SID, with the dose adjusted to maintain a tacrolimus trough level of 5–15 ng/ml in whole blood (measured by immunoassay, Abbott Architect i2000). Tacrolimus weaning was achieved by tapering down the dose by 20% each day over the course of 1 week. Tacrolimus was initially supplemented with Methotrexate (15 mg/m^2^ on day +1 and 10 mg/m^2^ on days +3 and +5 given intravenously). Two recipients with stable donor chimerism (33454 and 34666) received tacrolimus supplemented with post-transplant cyclophosphamide (40 mg/kg IV on day +3) and Belatacept (20 mg/kg IV on days +4, +7, +10, +14, +21, and +28, then every 2 weeks until initiation of tapering). On the days of cyclophosphamide treatment, animals received IV Normosol at ~15–20 ml/kg over 6–8 h to prevent sterile hemorrhagic cystitis. Animal 33455 received methylprednisolone (0.5 mg/kg SID intravenously) beginning at day +7 to treat symptoms of GvHD. Serum chemistry and complete blood counts were performed 2–3 times weekly or as clinically indicated. Animals were treated with intravenous fluids and nephrotoxic drug doses were adjusted as needed when azotemia was detected. Animals received appropriate supportive care, including analgesics, as clinically indicated under the supervision of an ONPRC veterinarian. Details of the anti-GvHD prophylaxis regimen specific to each recipient are shown in Supplementary Fig. 1.

### Donor hematopoietic stem cell transplant protocol

MHC-matched donor macaques received G-CSF (10 μg/kg subcutaneously, SID for five days) to expand stem cell numbers and AMD3100 (1 mg/kg subcutaneously, 12 h prior to apheresis) to mobilize stem cells into the peripheral blood. MCM were sedated with 10 mg/kg ketamine intramuscularly then anesthesia was maintained with isoflurane throughout the procedure. Following a surgical cut-down to the femoral artery, femoral arterial catheters (16 gauge, 8-inch intracath) were placed at the femoral triangle. One intravenous catheter was used as the blood return line from the leukapheresis machine and the other was used as a blood sampling port. Peripheral blood cells were then collected via leukapheresis using a Spectra Optia apheresis system. The Spectra Optia is designed for human donors with no less than 300 ml total blood volume (TBV). Due to the small size of MCM donors (~3–5 kg with TBV ~180–300 ml), 120 ml of ABO-matched, leukocyte-reduced priming blood was first primed through the Spectra Optia to ensure a safe donor hematocrit throughout the procedure. In addition, for animals below 300 ml TBV the Spectra Optia must be set to the highest inlet blood:acid citrate dextrose (ACD) ratio to ensure a sufficient flow rate for blood separation. Therefore, for animals below 300 ml TBV an external syringe pump containing ACD (solution A, Terumo BCT) was connected to a three-way stop cock directly anterior to the femoral catheter. ACD was infused into the line at 5 ml/h for the first 30 min of the procedure, followed by 2 ml/h thereafter. Blood samples were taken for iStat analysis every 30 min throughout the leukapheresis procedure to monitor blood chemistry. Supplemental calcium (4 mg/ml elemental calcium solution, initial rate of 40–45 ml/h) was infused intravenously to maintain blood-ionized calcium levels between 1.0 and 1.5 mmol/l. Body temperature was continuously monitored using an esophageal thermometer and a forced-air warming blanket was used to maintain body temperature. Red blood cells were removed from the leukapheresis product by ficoll density gradient and mononuclear cells subsequently washed twice in 50 ml PBS. Stem cells were isolated by staining with CD34-PE (Biolegend, Inc. – clone 561) and magnetic separation with anti-PE beads (Miltenyi, Inc.). CD34+ stem cell and CD3+T cell (BD Bioscience – clone SP34-2) frequencies in the positive and negative sorted fractions were assessed on a Becton Dickinson LSRII. Based on this data, CD34+ stem cell and CD3+ T cell doses were calculated and final infusion product was resuspended in 50 ml sterile PBS. Donor grafts were infused at a rate of 2 ml/min during which time recipient animals were monitored for immunological reactions.

### Donor chimerism measurement

SNP loci distinguishing donor and recipient genomes were identified using a custom 128 SNP array designed to genotype polymorphic loci distributed across the macaque genome adapted from Kanthaswamy et al.^42^. DNA (6.25 ng) from each macaque was enriched for target regions using TaqMan PreAmp Master Mix in conjunction with a custom TaqMan PreAMP pool, following manufacturer’s recommendations (Life Technologies). Enriched DNAs were diluted 1:10 in 1× TE buffer before being loaded onto OpenArray chips using the QuantStudio 12K Accufil System and assayed on a QuantStudio 12k Flex Real-Time PCR System (Life Techonologies). The resulting amplification and allele discrimination plots were visualized with TaqMan Genotyper v1.3 (Applied Biosystems, Inc.) to determine SNP genotypes. Donor engraftment was measured via two separate donor chimerism assays, a qualitative DNA surveyor assay performed in real time to inform clinical decisions (shown in Supplementary Fig. 3), and a post hoc quantitative deep sequencing assay (shown in Supplementary Fig. 3). In some cases, tissue mononuclear cells were sorted for CD45+, CD3+, or CD33+ cells by Miltenyi sorting prior to extracting genomic DNA for chimerism assays using APC-conjugated antibodies and anti-APC microbeads. For each animal, a panel of regions containing SNPs was amplified from genomic DNA, Sanger sequenced, and analyzed in Geneious (Supplementary Table 1). SNPs were chosen for donor-recipient pairs if heterozygous for the donor and homozygous for the recipient, or vice versa. To assess early chimerism in real time, gDNA was isolated from whole blood as stated previously. The appropriate SNP was amplified from the donor and recipient pre- and post-HSCT using the PCR conditions listed above. The amplicon was PCR purified and quantified by NanoDrop. The Surveyor Mutation Detection kit (IDT, cat. no. 706020) protocol was followed. Briefly, 400 ng of amplicon DNA from each time point in a total of 20 μl dH_2_O containing 2 ul 10× PCR buffer (ThermoFisher Scientific cat. no. 18067017) was hybridized by annealing slowly from 95 °C to 25 °C in a thermocycler to allow formation of a heteroduplex/homoduplex mixture at the SNP site. The mixture was treated with 2.5 μl MgCl_2_, 0.5 μl dH_2_O, 1 μl Surveyor Nuclease S, and 1 μl Surveyor Enhancer S and incubated at 42 °C for 35 min. A no-nuclease control was included. Samples were run on a 2% agarose gel, stained with SybrGold (Life Technologies), and photographed with a UV imager. Lanes containing SNP-heterozygous DNA have both a ~220 bp band (homoduplex) and a lower (~110 bp) band, comprising both halves of the heteroduplex amplicons cleaved by Surveyor Nuclease S. Lanes containing SNP-homozygous DNA have a single, uncleaved 220 bp band. Post-HSCT, the Surveyor assay can detect chimerism as low as 5%, though the assay is qualitative. To assess donor chimerism quantitatively post hoc, the SNP of interest from the recipient was amplified from gDNA from either whole blood or sorted cells (flow cytometric sorting described below) and prepared for deep sequencing with NEXTflex Rapid DNA Sequencing Bundle kit as described above, with the following modifications: 50–75 ng of each amplicon is used per reaction, in an equimolar ratio to any different SNPs occupying the same barcode. All reagents were used at one-third the volume and the final PCR (conditions in step C1) was eight cycles. The samples were run on an Illumina Miseq with a 2 × 250 kit and analyzed in Geneious. To determine percent chimerism, the calculations used were (2 × percentage of sequences containing the donor nucleotide) for a heterozygous donor/homozygous recipient, or 100 − (2 × percentage of sequences containing the recipient nucleotide) for a homozygous donor/heterozygous recipient, or the percentage of sequences containing the donor nucleotide for homozygous different donor and recipient. Initially, two or three SNPs were utilized and averaged to determine donor chimerism, but given the consistent accuracy of the assay, subsequent experiments utilized only one SNP.

### CMV monitoring and treatment

Cynomolgus macaque cytomegalovirus (CyCMV) levels in plasma were monitored via quantitative PCR. Plasma was concentrated by centrifugation at >20,000 rcf at 4 °C for 1 h, after which supernatant was removed to leave 200 μl volume. Viral nucleic acid was extracted from plasma using QIAamp MinElute Virus Spin Kit (Qiagen) according to manufacturer’s instructions. Quantitative PCR targeting a 121-bp region of CyCMV^43^ was carried out in 20 μl reactions by combining 5 μl of extracted viral nucleic acid, 10 μl TaqMan Fast Advanced Master Mix (Life Technologies), 1.5 μl of each 6 μM primer (forward: 5′-GGGCATTCTCAGGATCACAG-3′; reverse: 5′-TGACACCAAGAGGGTATGGG-3′), and 2 μl probe (5′-FAM-ACTCTGAAGACCACAAGGACCCACG-BHQ-3'). Sample reactions were run in duplicate in MicroAmp Fast Optical 96-well reaction plates (Applied Biosystems) alongside no template control reactions and standard reactions containing ten-fold serial dilutions of standard (10 copies to 10^7^ copies per reaction, in duplicate). Plasmid (pCEP4) containing the 121-bp CyCMV target region served as the standard. Plates were run on a StepOnePlus Real-Time PCR system (Applied Biosystems) with the following conditions: 95 °C 00:20, followed by 40 cycles of 95 °C 00:01, 60 °C 00:20. CyCMV genome copy number in each sample reaction was calculated according to the standard curve, and then adjusted for the proportion of viral nucleic acid added to the reaction (typically 1/6) and initial plasma volume (typically 1 ml) to obtain CyCMV genome copies per ml plasma. Sample reactions undetectable for CyCMV by quantitative PCR are reported as 0. When CyCMV levels >500 copies/ml were identified in plasma, treatment was initiated with 10 mg/kg oral valganciclovir SID, and subsequently escalated to 10 mg/kg oral valganciclovir BID until levels reached undetectable levels.

### CMV immunity

CMV-specific T cell responses were assessed by interferon-γ enzyme-linked immunospot assay (IFN-γ ELISPOT) using Monkey IFN-γ ELISPOT Plus ALP kit (Mabtech), following manufacturer’s instructions. ELISPOT assays were performed in duplicate, stimulating 100,000 peripheral blood mononuclear cells per well with rhesus CMV viral lysate and pools of 15-mer peptides (11 amino-acid overlap, 7–13 peptides per pool) spanning the RhCMV pp65a, pp65b, IE-1, and IE-2 proteins (Genbank Accession #AY186194). Negative control wells were incubated without antigen or with GagORF (pool of 15-mer peptides, 11 amino-acid overlap, spanning the entire SIVmac239 gag protein, Genbank Accession #M33262); positive control wells were incubated with Concanavalin A (ConA).

### GvHD grading

GvHD was monitored clinically using a previously described macaque GvHD clinical staging scale^10^. Briefly, this evaluation consisted of semi-quantitative individual scores assessing GvHD-mediated abnormalities of skin (presence and extent of skin rash), liver (extent of alterations in serum bilirubin levels), gastrointestinal tract (presence and extent of diarrhea), and activity (animal well-being and behavior). GvHD was also monitored by histopathology using paraffin-embedded tissues from organs collected at necropsy. Extent of GvHD was scored on hematoxylin/eosin stained slides by a GvHD histopathology expert blinded to the clinical history of the animals.

### Mixed lymphocyte reaction

Levels of T cell alloreactivity in recipient macaques were assessed via mixed lymphocyte reactions. Effector cells (PBMC or lymph node cells) were incubated in 1μM CFSE (Invitrogen) diluted in warm PBS for 10 min at 37 °C, quenched in cold R10 for 5 min, and washed twice with cold R10. CFSE-labeled effector cells were subsequently combined with lethally irradiated target cells (PBMC or bone marrow cells derived from donor or recipient pre-HSCT) in warm R10 in 48-well plates and cultured for 7 days at 37 °C. Effector cells cultured alone and cultured in Staphylococcus enterotoxin B (Toxin Technologies, Inc.) served as negative and positive controls, respectively. After 7 days of culture, cells were harvested, stained for CD3, CD4, and CD8, and viability (Live/Dead Fixable Yellow, Invitrogen), washed once with FACS buffer, fixed with 2% paraformaldehyde (PFA), and collected on a BD LSRII instrument. Levels of effector cell proliferation were assessed by frequency of CFSE-lo cells, gating on live CD3+ singlets.

### Flow cytometric analysis and sorting of immune system cells

Immune subset frequencies in HSCT recipients were monitored longitudinally by whole blood staining. EDTA-treated whole blood (50–100 μl) was washed twice with PBS and stained for CD45, CD3, CD20, CD4, CD8, CD14, CD28, and CD95, and viability (Live/dead Yellow Fixable, Invitrogen) at room temperature for 30 min. After staining, whole blood was resuspended in 1 ml FACS Lysing Solution (BD) to lyse red blood cells and fix remaining cells, incubated at room temperature for 8 min, washed three times with FACS buffer, and collected on a BD LSRII instrument. All flow cytometric analysis was performed using Flow Jo (Tree Star, Inc.). T cell frequency was defined as the percentage of CD20-CD3+ cells within the live CD45+ singlet population. Mobilized HSCT donor macaques were monitored for stem cells in peripheral blood by whole blood staining, conducted as described above, staining for CD45, CD34, and viability (Live/dead Yellow Fixable, Invitrogen). Stem cell frequency was defined as the percentage of CD34+ cells within the live CD45+ singlet population. Absolute T cell and stem counts were calculated by multiplying whole blood staining frequencies by the white blood cell count (WBC), as determined by complete blood count (CBC) performed on an additional aliquot of EDTA-treated whole blood.

Levels of T cell proliferation in necropsy tissues were assessed by intracellular Ki67 staining. Tissues were processed into mononuclear cell preparations, as previously described^44^, washed once in PBS, and stained for CD45, CD3, CD20 and CD8b, and viability (Live/dead Yellow Fixable, Invitrogen) at room temperature for 30 min. Cells were subsequently washed once with FACS buffer, fixed in 2% PFA, permeabilized with saponin buffer (1× PBS with 0.5% saponin and 10% FBS), and stained intracellularly for Ki67 overnight at room temperature. The following day, cells were washed with saponin buffer and collected on a BD LSRII instrument. Frequencies of Ki67+ T cells were determined within the live, CD45+, CD20−, CD3+ singlet population, graphing CD8b vs. Ki67 (CD4+ T cells defined as CD8b−). Frequencies of HLA-DR+ cells were identified in a similar manner, but with HLA-DR included the surface stain and no subsequent permeabilization.

To assess levels of donor chimerism in specific immune cell subsets, whole blood cells or lymph node biopsy mononuclear cells were sorted on a BD Aria instrument. Whole blood was treated with ACK Lysing Solution (Fisher Scientific) to lyse red blood cells, incubated at 5 min at room temperature, and washed twice with PBS. ACK-treated whole blood or lymph node mononuclear cells were stained for 30 min at room temperature and subsequently washed with FACS buffer prior to sorting. Cells were kept at 4 °C until sorting. For monitoring whole blood T cell and granulocyte donor chimerism levels, cells were stained for CD3 and CD45, and for viability in stains using thawed cells. For comprehensive assessment of whole blood and lymph node immune subset donor chimerism levels, the following staining panels were used: Panel 1 (T follicular helper cells), CD45, CD3, CD4, CD95, ICOS or CXCR5, PD-1, Live/dead fixable yellow; Panel 2 (NK, T, B, monocytes), CD45, CD3, CD14, CD20, CD8, Live/dead fixable yellow; Panel 3 (T cell subsets), CD45, CD3, CD4, CD8, CD28, CD95, Live/dead fixable yellow. Immune subsets within the live singlets population were defined and sorted as follows: bulk T cells (CD45-hi, SSC-lo, CD3+), granulocytes (CD45-lo/mid, SSC-hi), T follicular helper cells (CD45-hi, SSC-lo, CD3+, CD4+, CD95+, PD1-hi, ICOS+, or CXCR5+), NK cells (CD45-hi, SSC-lo, CD14−, CD20−, CD3−, CD8+), B cells (CD45-hi, SSC-lo, CD14−, CD20+, CD3−), monocytes (CD45-mid, SSC-mid/high, CD3−, CD20−, CD14+), bulk CD4+ T cells (CD45-hi, SSC-lo, CD3+, CD4+, CD8−), and bulk CD8+ T cells (CD45-hi, SSC-lo, CD3+, CD4−, CD8+). Memory T cell subsets within bulk CD4+ or CD8+ T cell populations were defined and sorted as follows: naive (CD95−, CD28+), central memory (CD95+, CD28+), and effector memory (CD95+, CD28−). For each sorted population, 15,000–150,000 cells (>95% pure) were collected and used for genomic DNA extraction, with the exception of lymph node T follicular helper cells, NK cells, and effector memory T cells, for which 4,000–15,000 cells were collected.

### Antibodies

The following monoclonal antibodies were used in these studies: (a) from BD Biosciences, D058-1283 (CD45; APC, PE-Cy7), SP34 (CD3; FITC), SP34-2 (CD3; Alexa700, PacBlu), 2H7 (CD20; APC-H7), L200 (CD4; PerCP-Cy5.5, Alexa700), RPA-T8 (CD8; PacBlu), SK1 (CD8, TruRed), 28.2 (CD28; PE), DX2 (CD95; FITC, PE-Cy7), MU5UBEE (CD185/CXCR5; PeCy7), (b) from Beckman Coulter, RMO52 (CD14; ECD), 2ST8.5H7 (CD8b; PE), (c) from Miltenyi Biotec, M-T466 (CD4; APC), (d) from eBioscience, ISA-3 (ICOS/CD278, FITC), J105 (PD1/CD279, eFluor710), (e) from Biolegend, 561 (CD34; PE, APC), and (f) from Invitrogen, FN18 (CD3; FITC).

### Code availability

The custom MHC analysis pipeline written by B.N.B. is available through DISCVR-Seq (https://github.com/bbimber/discvr-seq), as a module for LabKey Server, an open-source platform for the management of scientific data.

### Data availability

The data that support the findings of this study are available from the corresponding author upon request

## Electronic supplementary material

Supplementary information

Peer review file
