# Supplementary material for: Allogeneic stem cell transplantation in fully MHC-matched Mauritian cynomolgus macaques recapitulates diverse human clinical outcomes
Source: Nat Commun. 2017 Nov 10;8:1418. doi: 10.1038/s41467-017-01631-z (PMC5681693; doi:10.1038/s41467-017-01631-z)
Supplement: Supplementary file 1 — Supplementary information [file 41467_2017_1631_MOESM1_ESM.pdf]

Recipient ID: 32851

					Pre-transplant						HSCT	Post-transplant																	
Treatment	Purpose	Dose	Timing	Route	-6	-5	-4	-3	-2	-1	0	1	2	3	4	5	6	7	8	9	10	11	12	13	14	…	21	…	25
Levetiracetam	Anti-convulsion	13 mg/kg	SID	IV																									
Busulfan	Immune conditioning	4 mg/kg	SID	IV																									
Fludarabine	Immune conditioning	30 mg/m^2	SID	IV																									
Total Body Irradiation	Immune conditioning	2 Gy @ 15cGy/min	-	-																									
Cefazolin	Antibiotic	25 mg/kg	BID	IV																									
Filgrastim	Immune reconstitution	0.01 mg/kg	SID	SC																									
Tacrolimus	GVHD prophylaxis	0.08 mg/kg/day	Drip	IV																									
Methotrexate	GVHD prophylaxis	10 or 15* mg/m^2	SID	IV								*																	

Recipient ID: 32846 HSCT #1

					Pre-transplant						HSCT	Post-transplant																	
Treatment	Purpose	Dose	Timing	Route	-6	-5	-4	-3	-2	-1	0	1	2	3	4	5	6	7	8	9	10	11	12	13	14	…	21	…	28
Levetiracetam	Anti-convulsion	13 mg/kg	SID	IV																									
Busulfan	Immune conditioning	3.2 mg/kg	SID	IV																									
Fludarabine	Immune conditioning	30 mg/m^2	SID	IV																									
Total Body Irradiation	Immune conditioning	2 Gy @ 15cGy/min	-	-																									
Cefazolin	Antibiotic	25 mg/kg	BID	IV																									
Pegfilgrastim	Immune reconstitution	0.3 mg/kg	SID	SC																									
Tacrolimus	GVHD prophylaxis	0.08 mg/kg/day	Drip	IV																									
Methotrexate	GVHD prophylaxis	10 or 15* mg/m^2	SID	IV								*																	

Recipient ID: 32849

					Pre-transplant							HSCT	Post-transplant																	
Treatment	Purpose	Dose	Timing	Route	-13	-6	-5	-4	-3	-2	-1	0	1	2	3	4	5	6	7	8	9	10	11	12	13	14	…	21	…	27
Levetiracetam	Anti-convulsion	13 mg/kg	SID	IV																										
CD8 depleting antibody (cM-T807)	Immune conditioning	10 mg/kg		SC																										
Busulfan	Immune conditioning	6 mg/kg	SID	IV																										
Fludarabine	Immune conditioning	30 mg/m^2	SID	IV																										
Total Body Irradiation	Immune conditioning	3 Gy @ 20cGy/min	-	-																										
Cefazolin	Antibiotic	25 mg/kg	BID	IV																										
Pegfilgrastim	Immune reconstitution	0.3 mg/kg	SID	SC																										
Tacrolimus	GVHD prophylaxis	0.12 mg/kg/day	Drip	IV																										
Methotrexate	GVHD prophylaxis	10 or 15* mg/m^2	SID	IV									*																	

Recipient ID: 32846 HSCT #2

					Pre-transplant						HSCT	Post-transplant																
Treatment	Purpose	Dose	Timing	Route	-6	-5	-4	-3	-2	-1	0	1	2	3	4	5	6	7	8	9	10	11	12	13	14	15	16	17
Levetiracetam	Anti-convulsion	13 mg/kg	SID	IV																								
CD3-immunotoxin	Immune conditioning	0.025mg/kg	BID	IV																								
Busulfan	Immune conditioning	6 mg/kg	SID	IV																								
Total Body Irradiation	Immune conditioning	3 Gy @ 20cGy/min	-	-																								
Cefazolin	Antibiotic	25 mg/kg	BID	IV																								
Filgrastim	Immune reconstitution	0.01 mg/kg	SID	SC																								
Tacrolimus	GVHD prophylaxis	0.16 mg/kg/day	Drip	IV																								
Methylprednisolone	GVHD prophylaxis	0.5 mg/kg	SID	IV																								
Methotrexate	GVHD prophylaxis	10 or 15* mg/m^2	SID	IV								*																

Recipient ID: 33450

					Pre-transplant						HSCT	Post-transplant															
Treatment	Purpose	Dose	Timing	Route	-6	-5	-4	-3	-2	-1	0	1	2	3	4	5	6	7	8	9	10	11	12	13	14	…	17
CD3-immunotoxin	Immune conditioning	0.025mg/kg	BID	IV																							
Busulfan	Immune conditioning	6 mg/kg	SID	IV																							
Fludarabine	Immune conditioning	30 mg/m^2	SID	IV																							
Total Body Irradiation	Immune conditioning	3 Gy @ 20cGy/min	-	-																							
Cefazolin	Antibiotic	25 mg/kg	BID	IV																							
Pegfilgrastim	Immune reconstitution	0.3 mg/kg	SID	SC																							
Tacrolimus	GVHD prophylaxis	0.12 mg/kg/day	Drip	IV																							
Methotrexate	GVHD prophylaxis	10 or 15* mg/m^2	SID	IV								*															

Recipient ID: 33455

					Pre-transplant						HSCT	Post-transplant															
Treatment	Purpose	Dose	Timing	Route	-6	-5	-4	-3	-2	-1	0	1	2	3	4	5	6	7	8	9	10	11	12	13	14	15	16
CD3-immunotoxin	Immune conditioning	0.025mg/kg	BID	IV																							
Busulfan	Immune conditioning	6 mg/kg	SID	IV																							
Total Body Irradiation	Immune conditioning	4 Gy @ 20cGy/min	-	-																							
Cefazolin	Antibiotic	25 mg/kg	BID	IV																							
Tacrolimus	GVHD prophylaxis	0.16 mg/kg/day	Drip	IV																							
Methylprednisolone	GVHD prophylaxis	0.5 mg/kg	SID	IV																							
Methotrexate	GVHD prophylaxis	10 or 15* mg/m^2	SID	IV								*															

Recipient ID: 33454

					Pre-transplant						HSCT	Post-transplant																												
Treatment	Purpose	Dose	Timing	Route	-6	-5	-4	-3	-2	-1	0	1	2	3	4	5	6	7	8	9	10	11	12	13	14	…	21	…	28	29	30	…	35	…	42	49	56	63	…	70
CD3-immunotoxin	Immune conditioning	0.025mg/kg	BID	IV																																				
Busulfan	Immune conditioning	6 mg/kg	SID	IV																																				
Total Body Irradiation	Immune conditioning	2 Gy @ 20cGy/min	-	-																																				
Polymyxin B	Gut decontamination	1x10^6 IU	SID	PO																																				
Neomycin sulfate	Gut decontamination	500 mg	SID	PO																																				
Cefazolin	Antibiotic	25 mg/kg	BID	IV																																				
Tacrolimus	GVHD prophylaxis	0.16 mg/kg/day	Drip	IV																																				
		0.04 mg/kg	SID	IM																																			X	X
Belatacept	GVHD prophylaxis	20 mg/kg	SID	IV																																				
Cyclophosphamide	GVHD prophylaxis	40 mg/kg	SID	IV																																				

Recipient ID: 34666

					Pre-transplant						HSCT	Post-transplant																											
Treatment	Purpose	Dose	Timing	Route	-6	-5	-4	-3	-2	-1	0	1	2	3	4	5	6	7	8	9	10	11	12	13	14	…	21	…	28	29	30	…	35	…	42	…	69	…	76
CD3-immunotoxin	Immune conditioning	0.025mg/kg	BID	IV																																			
Busulfan	Immune conditioning	6 mg/kg	SID	IV																																			
Total Body Irradiation	Immune conditioning	2 Gy @ 20cGy/min	-	-																																			
Polymixin B	Gut decontamination	1x10^6 IU	SID	PO																																			
Neomycin sulfate	Gut decontamination	500 mg	SID	PO																																			
Cefazolin	Antibiotic	25 mg/kg	BID	IV																																			
Tacrolimus	GVHD prophylaxis	0.16 mg/kg/day	Drip	IV																																			
		0.04 mg/kg	SID	IM																																		X	X
Belatacept	GVHD prophylaxis	20 mg/kg	SID	IV																																			
Cyclophosphamide	GVHD prophylaxis	40 mg/kg	SID	IV																																			

Supplementary figure 1: Recipient drug regimens.

Tables denote purpose, dose, timing, and route of drugs administered to each recipient macaque pre- and post-HSCT. Black asterisks (*) indicate higher dose of methotrexate (15 mg/m^2). Black X's indicate period of tacrolimus dose tapering.

a

Treatment/Procedure	Purpose	Dose	Timing	Route	Pre-leukapheresis -4	-3	-2	-1	0
Filgrastim	Stem cell mobilization	0.01 mg/kg	SID	SC					
Plerixafor	Stem cell mobilization	1 mg/kg	SID	SC					
Leukapheresis	Stem cell collection	-	-	IV					

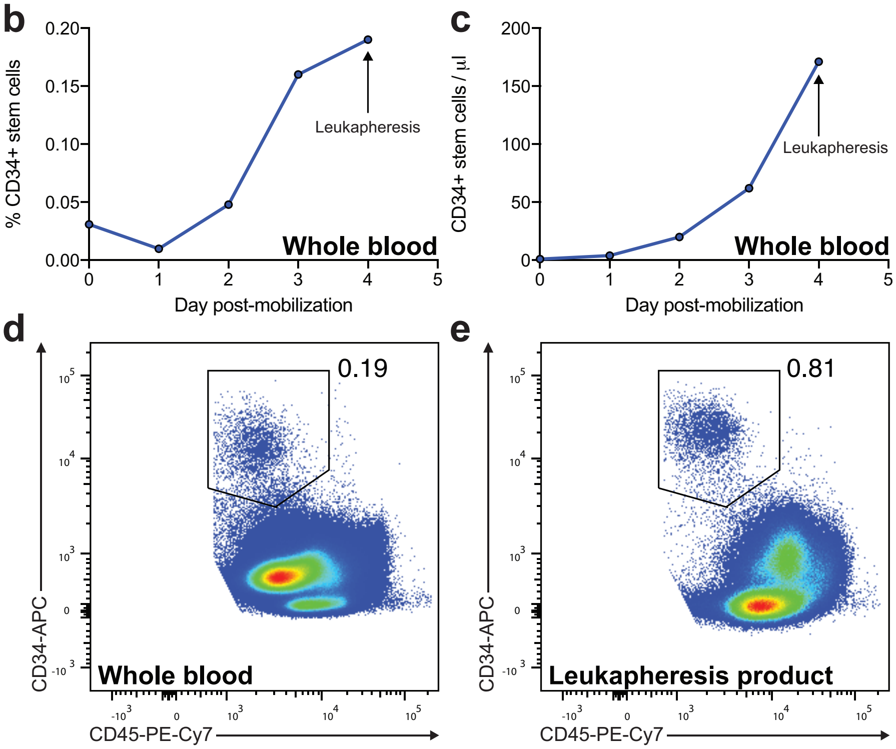

Supplementary figure 2: Donor mobilization drug regimen and leukapheresis product.
(a) Table denotes purpose, dose, timing, and route of drugs administered to each donor macaque prior to leukapheresis. Leukapheresis was performed 12-24 hours after plerixafor administration. **(b,c)** Longitudinal frequencies **(b)** and absolute counts **(c)** of CD34+ stem cells in peripheral blood of donor macaque 32850. **(d,e)** Representative flow cytometric plots of CD34+ stem cell frequencies in whole blood at the time of leukapheresis **(d)** and in leukapheresis product **(e)** for donor macaque 32850. Plots are gated on live CD45+ singlets.

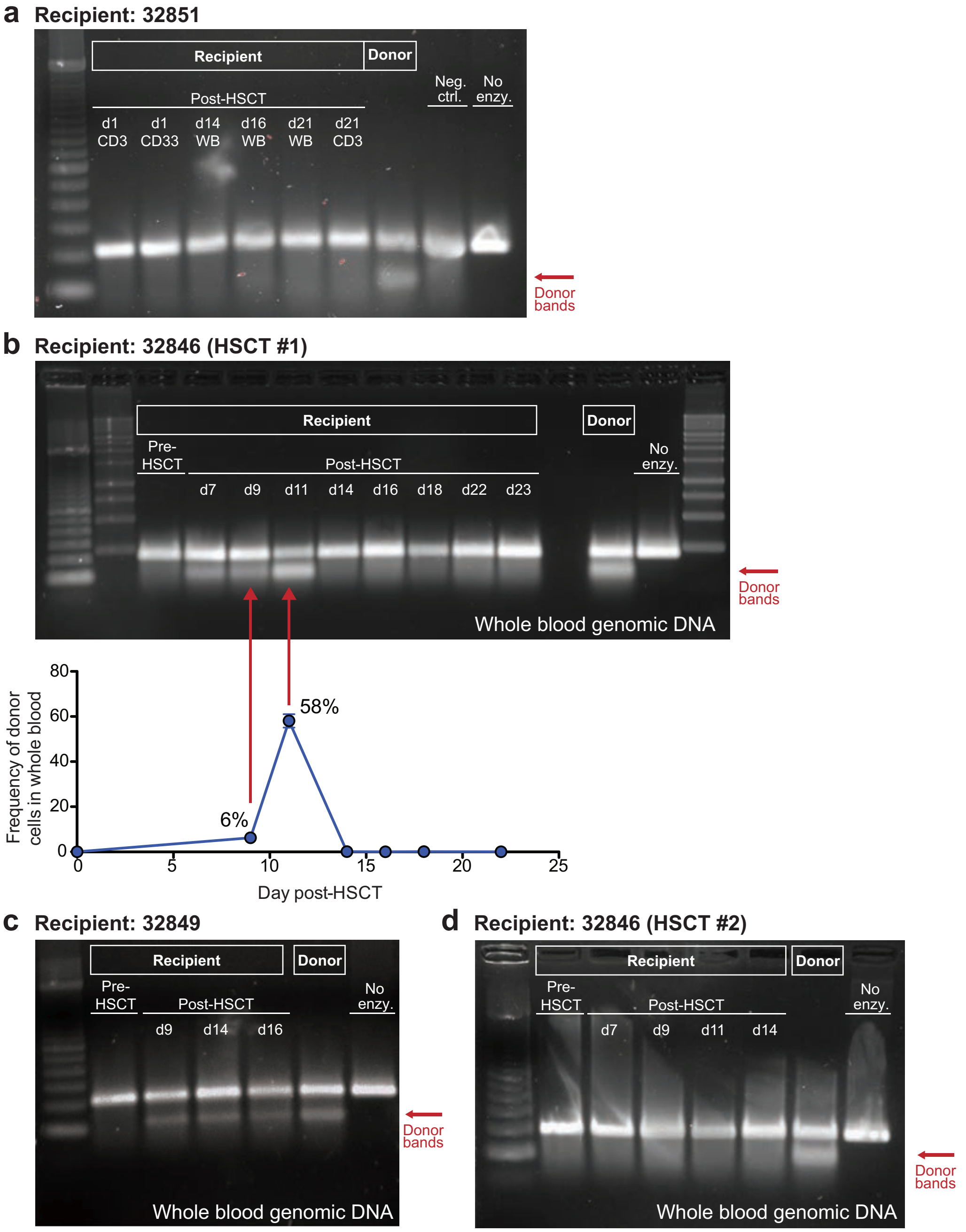

Supplementary figure 3: Chimerism assays for recipients with engraftment failure.
Donor chimerism detection assays for recipient macaques 32851 **(a)**, 32846 **(b,d)**, and 32849 **(c)**. **(a, b top panel, c, d)** Agarose gel images show longitudinal Surveyor assay detection of donor-derived genomic DNA in recipient macaques. Surveyor of SNP 5 (donor is heterozygous A/G, recipient is homozygous G/G) is shown in **(a)**. Surveyor of SNP 5 (donor is heterozygous A/G, recipient is homozygous A/A) is shown in **(b top panel** HSCT #1**, d** HSCT #2**)**. Surveyor of SNP 4 (donor is heterozygous A/G, recipient is homozygous G/G) is shown in **(c)**. Upper bands represent uncleaved homoduplex amplicons, while lower bands (red arrows) represent heteroduplex amplicons cleaved by Surveyor Nuclease S at the SNP site. No-enzyme controls were performed with SNP-heterozygous amplicons. Where indicated, whole blood (WB) cells were sorted for CD3+ T cells or CD33+ myeloid cells prior to genomic DNA extraction. **(b bottom panel)** Graph displays longitudinal donor chimerism levels in recipient macaque 32846 as measured by Illumina sequencing genomic DNA across three SNPs, with time points corresponding to Surveyor-based detection (red arrows).

a

CD3ε	MHC haplotypes	Amino acid position		
		35	45	50
FN18-susceptible		V	E	R
MCM 1	M3/M4	V	E	R
MCM 2	M2/M3	V	E	R
MCM 3	M4/M4	V	E	R
MCM 4	M1/M2	V	E	R
MCM 5	M1/M1	V	E	R
MCM 6	M5/M6	V	E	R
MCM 7	M2/M5	V	E	R
MCM 8	M4/M6	V	E	R
MCM 9	M3/M6	V	E	R
MCM 10	M3/M5	V	E	R
32843	M2/M3	V	E	R
32846	M2/M3	V	E	R
33452	M1/M3	V	E	R
33450	M1/M3	V	E	R
33454	M2/M4	V	E	R
33455	M1/M3	V	E	R
33456	M2/M6	V	E	R
33460	M2/M4	V	E	R
33461	M2/M4	V	E	R
34666	M2/M6	V	E	R
35132	M1/M3	V	E	R
35133	M1/M3	V	E	R

b

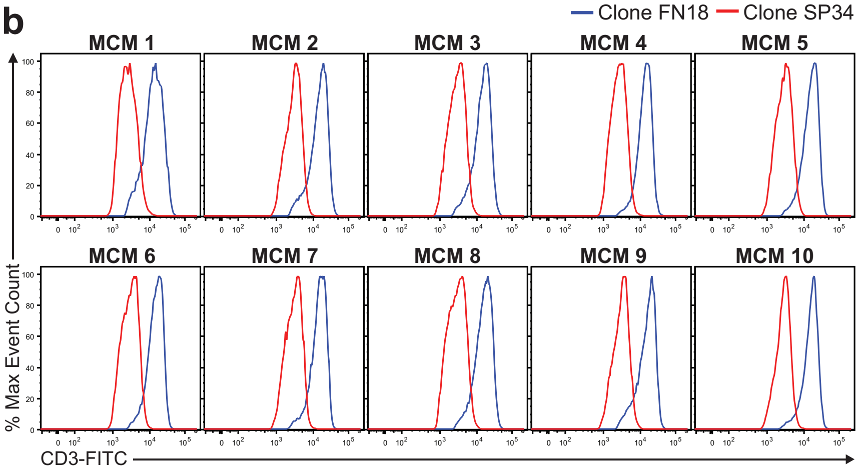

Supplementary figure 4: CD3 typing for CD3-immunotoxin susceptibility.

(a) Table summarizes CD3ε Sanger sequencing results for 22 Mauritian cynomolgus macaques of diverse MHC types. Residues corresponding to anti-CD3 mAb clone FN18 susceptibility[20] are shown in blue for reference. **(b)** Representative histogram overlays of anti-CD3 mAb clone FN18 (blue) versus clone SP34 (red) staining of PBMC from 10 Mauritian cynomolgus macaques shown in part (a). Histograms are gated on live CD3+ singlets.

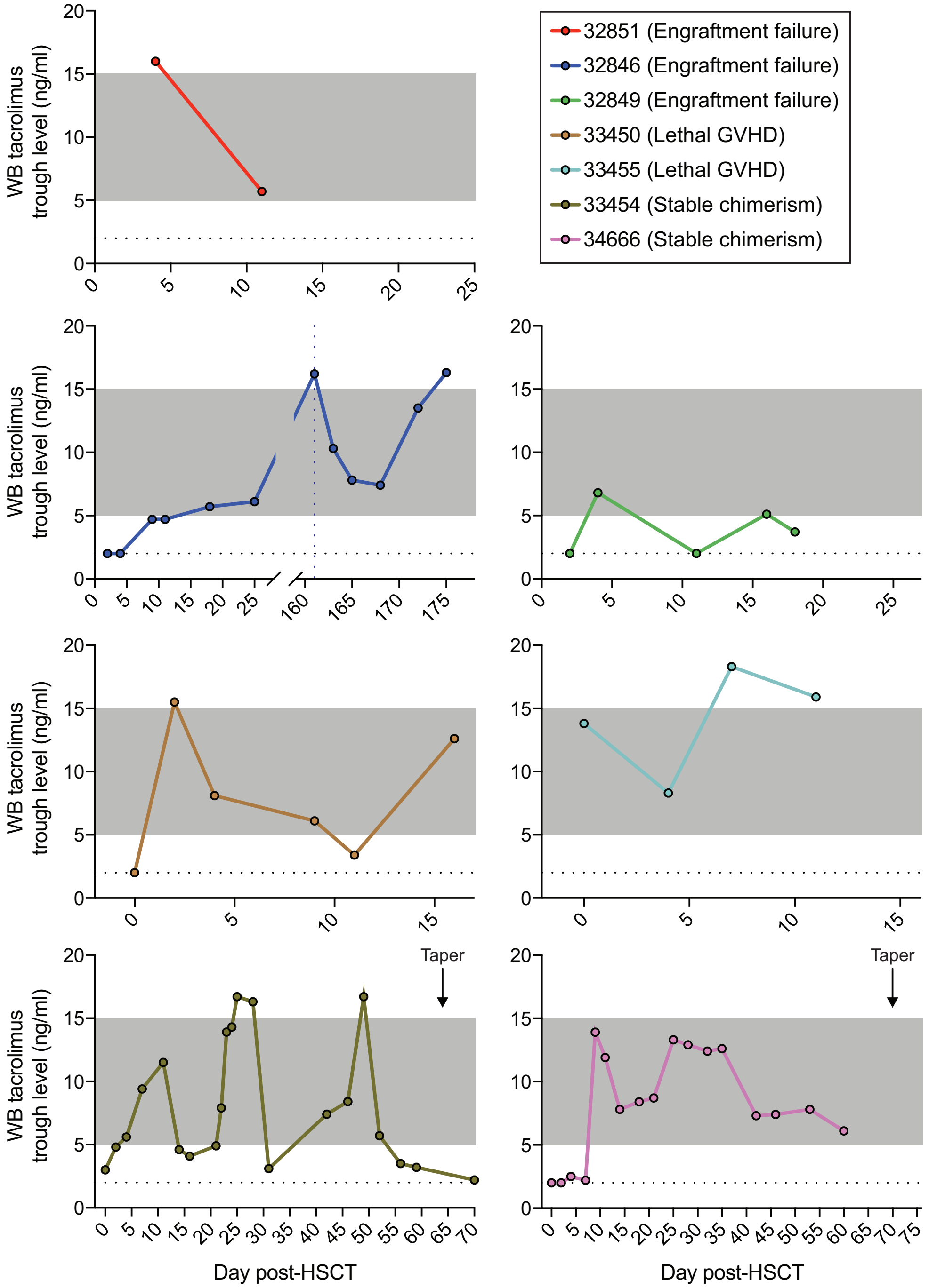

Supplementary figure 5: Tacrolimus trough levels in recipient macaques.
Tacrolimus trough levels in each recipient macaque during the tacrolimus treatment period. The trough level is measured from whole blood taken 30 to 60 minutes prior to administering the next dose. Shaded gray boxes indicated the desired therapeutic range. Dotted blue line indicates second HSCT of 32846. Arrows indicate time points at which taper of tacrolimus treatment was initiated.

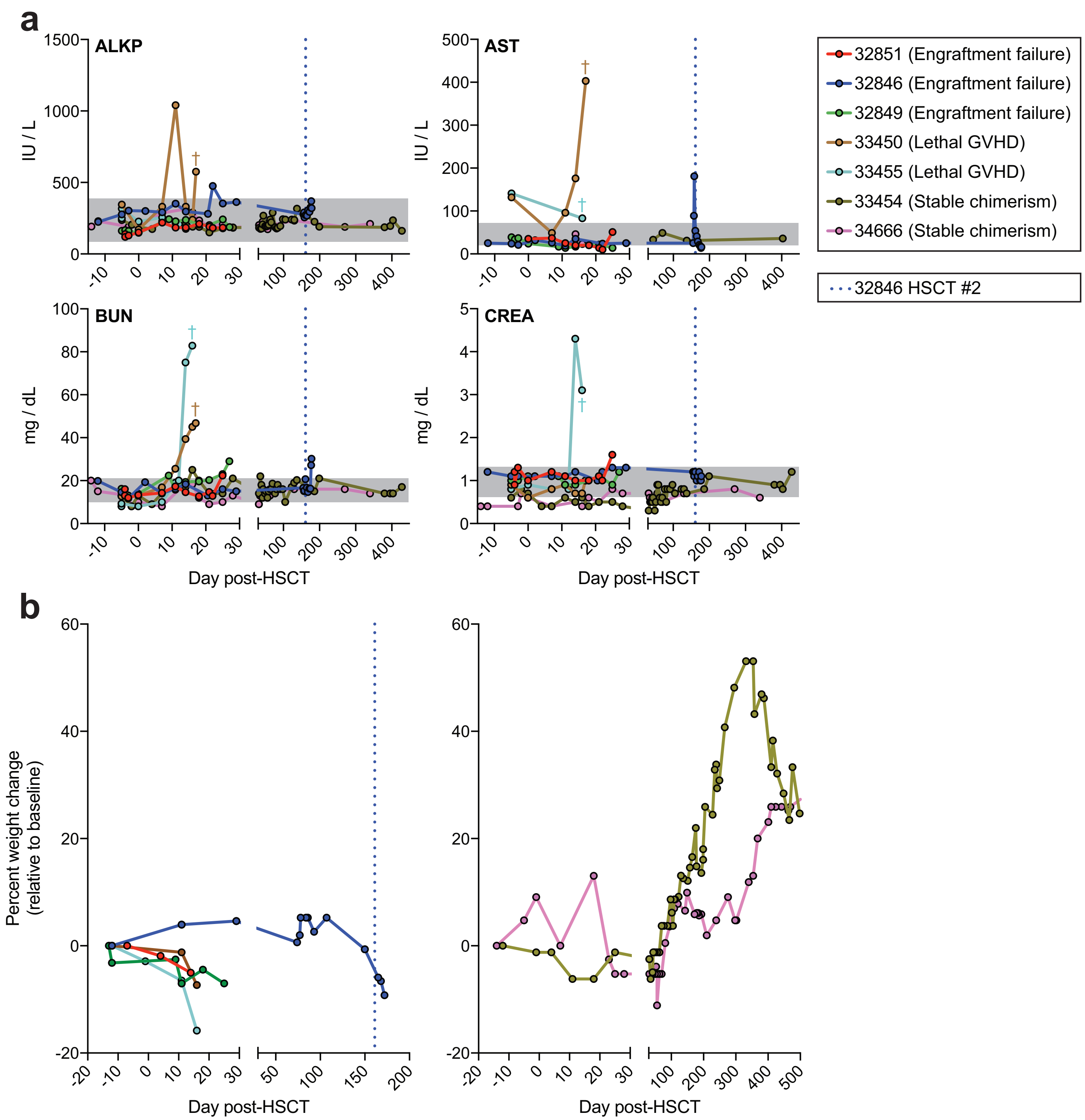

Supplementary figure 6: Recipient macaque serum chemistries and weight changes.
(a) Serum levels of alkaline phosphatase (ALKP), aspartate aminotransferase (AST), blood urea nitrogen (BUN), and creatinine (CREA) in recipient macaques. Shaded gray boxes indicated the normal ranges. Dotted blue line indicates second HSCT of 32846. **(b)** Percent weight changes from baseline in recipient macaques that experienced engraftment failure or GVHD (left) or stable donor chimerism (right). Baseline weight taken prior to conditioning.

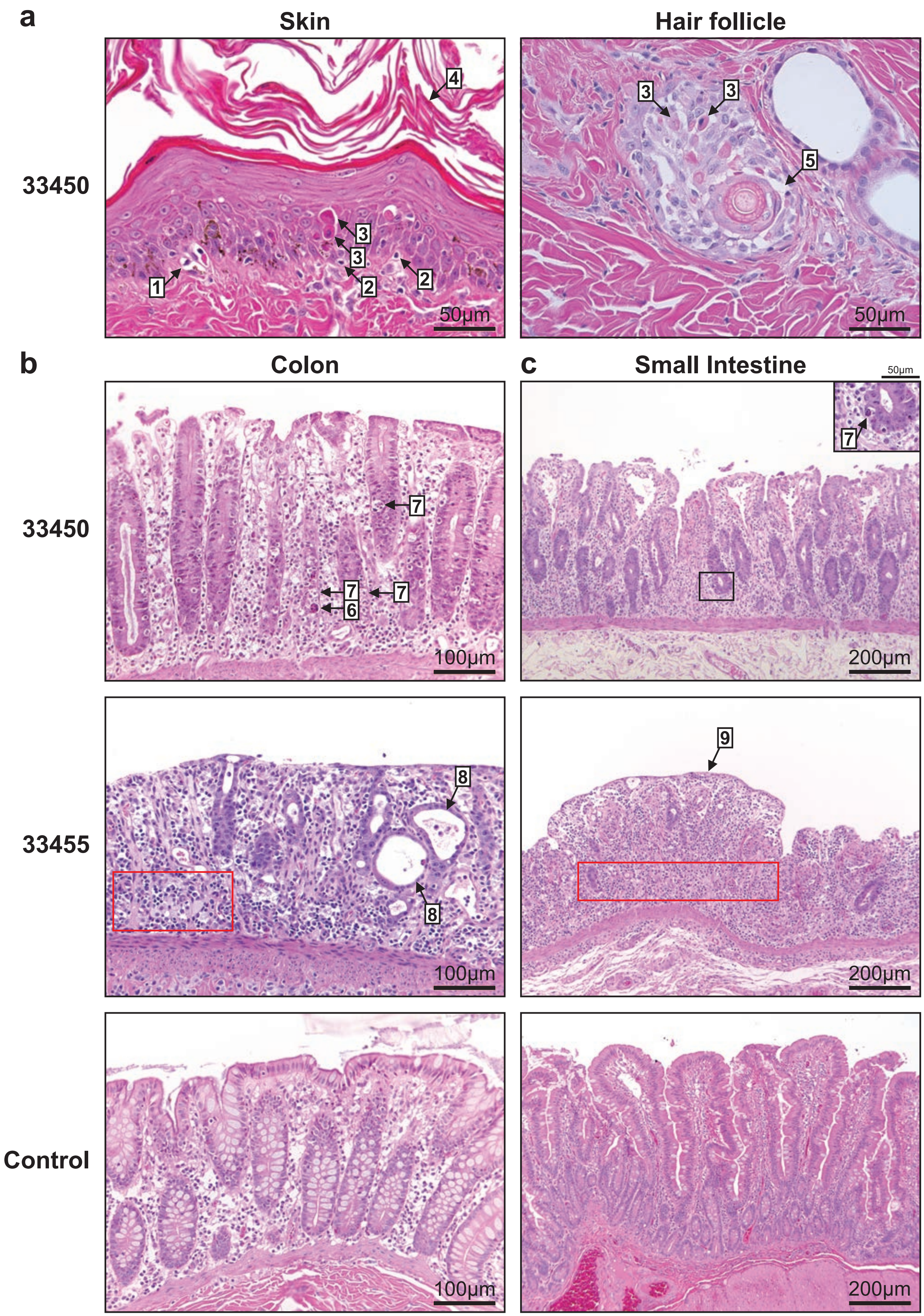

a
Skin
Hair follicle
33450
1
2
3
4
5
50µm
b
Colon
c
Small Intestine
6
7
8
9
100µm
200µm
33455
Control

Supplementary figure 7: Histopathology of recipients with lethal GvHD.
(a,b) Photomicrographs of hematoxylin and eosin (H&E)-stained sections of skin **(a)**, colon **(b)**, and small intestine **(c)** from recipient macaques 33450 and 33455. Scale for each image is denoted at bottom right. **(a, left)** Skin of 33450 exhibits an interface dermatitis characterized by vacuolar change of the basal cell epidermal layer (1), a sparse population of infiltrating lymphocytes (2), and apoptotic keratinocytes (3). The overlying stratum corneum is thickened and demonstrates orthokeratotic hyperkeratosis (4). **(a, right)** Multiple apoptotic keratinocytes (3) and epithelial vacuolar change (5) are also present in the hair follicle. **(b)** 33450 colon **(top)**: The lamina propria is expanded by a mixed inflammatory infiltrate of lymphocytes and plasma cells. Crypt epithelium is hyperplastic, and multifocal crypt epithelial cells are apoptotic (6) or exploded with karyorrhectic debris (popcorn cells, 7). 33455 colon **(middle)**: the lamina propria is expanded by a mixed inflammatory infiltrate of lymphocytes and plasma cells, with dilated crypts (8) and loss of crypt epithelium (red box), and erosion of surface enterocytes. **(c)** 33450 small intestine **(top)**: There is marked loss of crypts, and the lining epithelium of remaining crypts is hyperplastic with multifocal apoptosis. Villi are blunted and fused and the surface enterocytes are attenuated, rounded and occasionally form protruding epithelial tags. The lamina propria is expanded by lymphocytes, macrophages and few plasma cells and neutrophils. The epithelial cells lining the crypts are multifocally apoptotic. Inset: Apoptosis of crypt epithelium with “popcorn” lesions containing karyorrhectic and cellular debris (7). 33455 small intestine **(middle)**: The normal microarchitecture is severely altered with almost complete loss of villi (9), erosions, surface enterocyte attenuation and extensive crypt destruction and loss (red box). The remaining crypts are hyperplastic with multifocal apoptosis. Plasma cells, lymphocytes, and fewer macrophages and neutrophils expand the lamina propria. Sections of colon **(b, bottom)** and small intestine **(c, bottom)** from a healthy control Mauritian cynomolgus macaque are shown for reference.

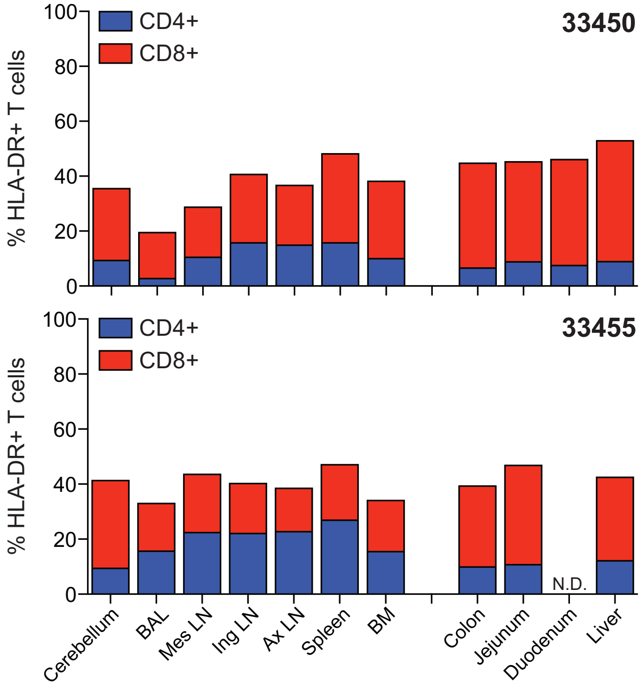

Supplementary figure 8: HLA-DR expression on T cells in tissues of recipients with lethal GvHD. Frequencies of HLA-DR+ T cells in tissues at time of necropsy, as determined by surface HLA-DR flow cytometric staining, in recipient macaques 33450 and 33455.

a **Recipient: 33454**

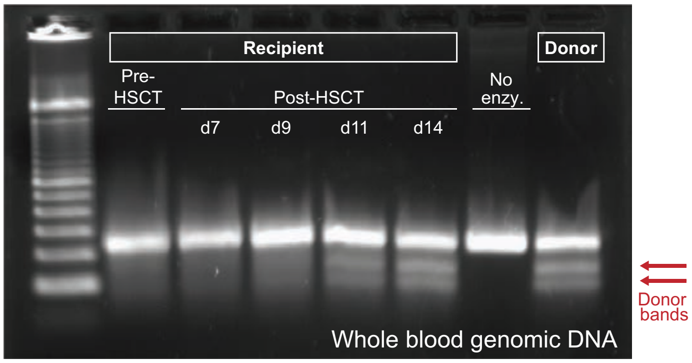

b **Recipient: 34666**

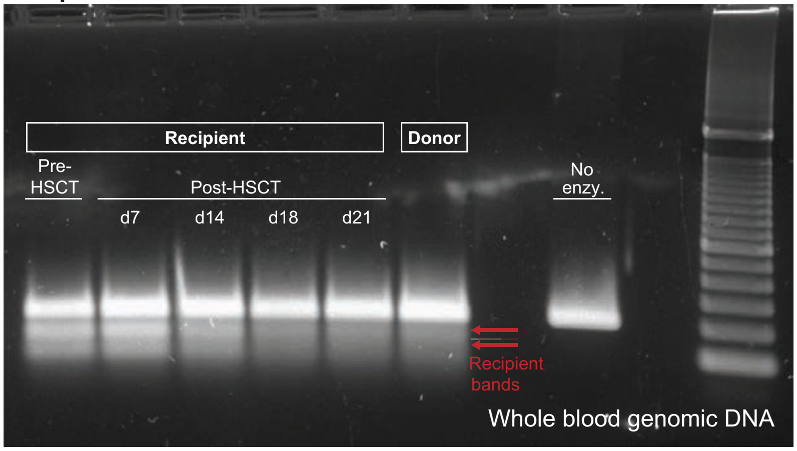

Supplementary figure 9: Surveyor chimerism assays for recipients with stable engraftment. Surveyor donor chimerism detection assays for recipient macaques 33454 **(a)** and 34666 **(b)**. **(a,b)** Agarose gel images show longitudinal Surveyor assay detection of donor-derived genomic DNA in recipient macaques. Surveyor of SNP 10 (donor is heterozygous T/C, recipient is homozygous C/C) is shown in **(a)**. Surveyor of SNP 1 (donor is homozygous C/C, recipient is heterozygous C/A) is shown in **(b)**. Upper bands represent uncleaved homoduplex amplicons, while lower bands (red arrows) represent heteroduplex amplicons cleaved by Surveyor Nuclease S at the SNP site. No-enzyme controls were performed with SNP-heterozygous amplicons.

Clinical GvHD Scores

	Day post-HSCT	Skin	Liver	GIT	Activity
33454	7	0	0	0	3
	11	0	0	0	3
	14	0	0	0	2
	18	0	0	1	3
	21	0	0	0	3
	25	0	0	0	3
	28	0	0	1	3
	30	0	0	0	3
	32	1	0	1	3
	36	1	0	1	3
	42	1	0	0	3
	49	1	0	0	3
	56	1	0	0	3
34666	4	0	No Chem	0	3
	7	0	0	0	3
	11	0	0	0	3
	14	0	0	0	3
	18	0	0	0	3
	21	0	0	0	3
	25	1	0	0	3
	28	0	0	1	3
	30	0	No Chem	0	3
	32	0	0	0	3
	35	0	0	0	3
	42	0	0	0	3
	49	0	0	0	3
	56	0	0	0	3
	63	0	0	0	3
	70	0	0	0	3

Supplementary figure 10: Clinical GvHD scoring of recipients with stable engraftment.
Longitudinal clinical GvHD scoring of recipient macaques 33454 and 34666. Scoring criteria described in Materials & Methods section. Note healthy macaques not undergoing HSCT occasionally experience pruritus and rash consistent with skin GvHD score of 1, as well as mounding stool and diarrhea consistent with GIT GvHD score of 1.

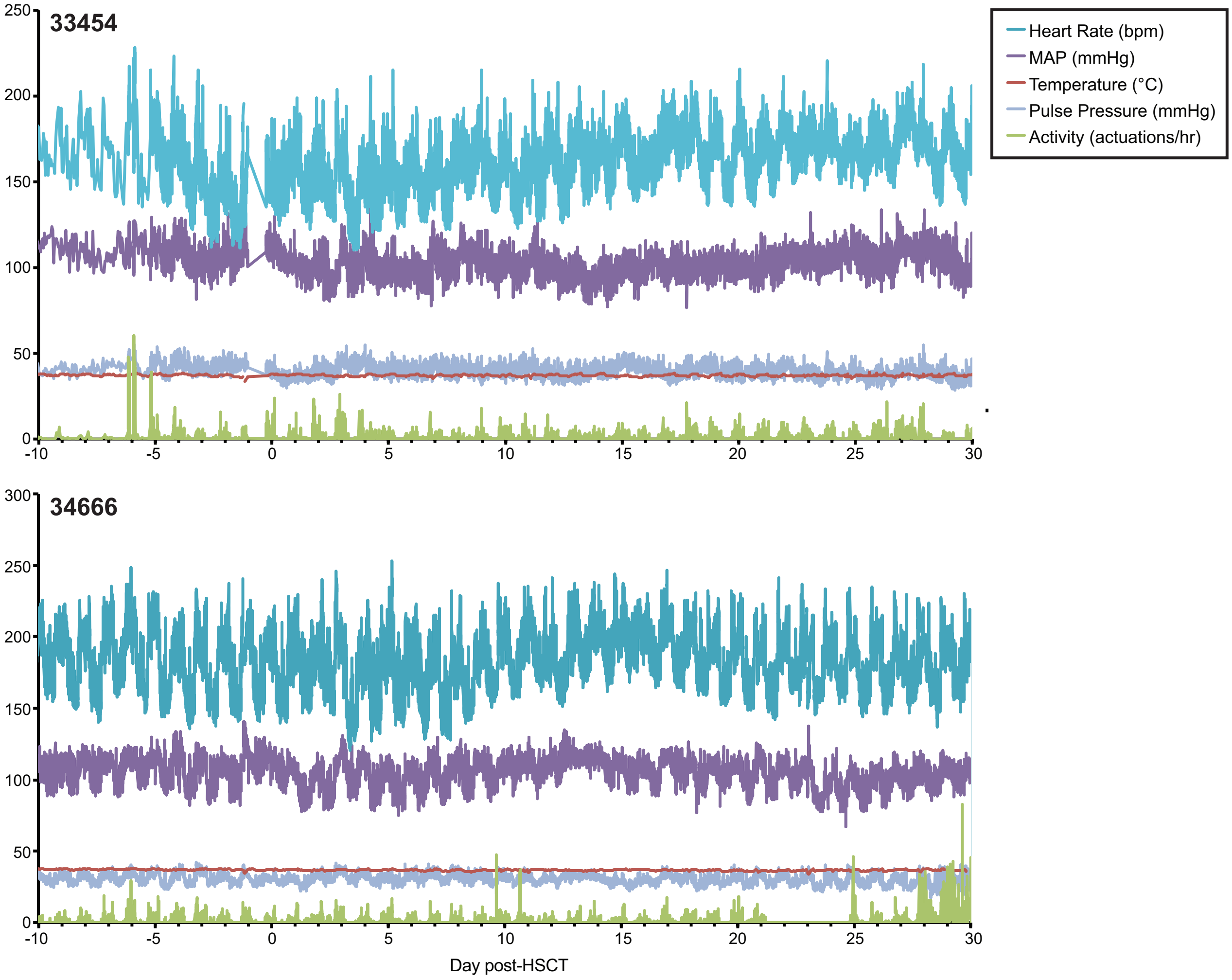

Supplementary figure 11: Telemetry measurements in recipients with stable engraftment.
Longitudinal telemetry measurements of heart rate, mean arterial pressure (MAP), temperature, pulse pressure, and activity in recipient macaques 33454 **(top)** and 34666 **(bottom)**. No significant changes in these measurements were observed post-HSCT.

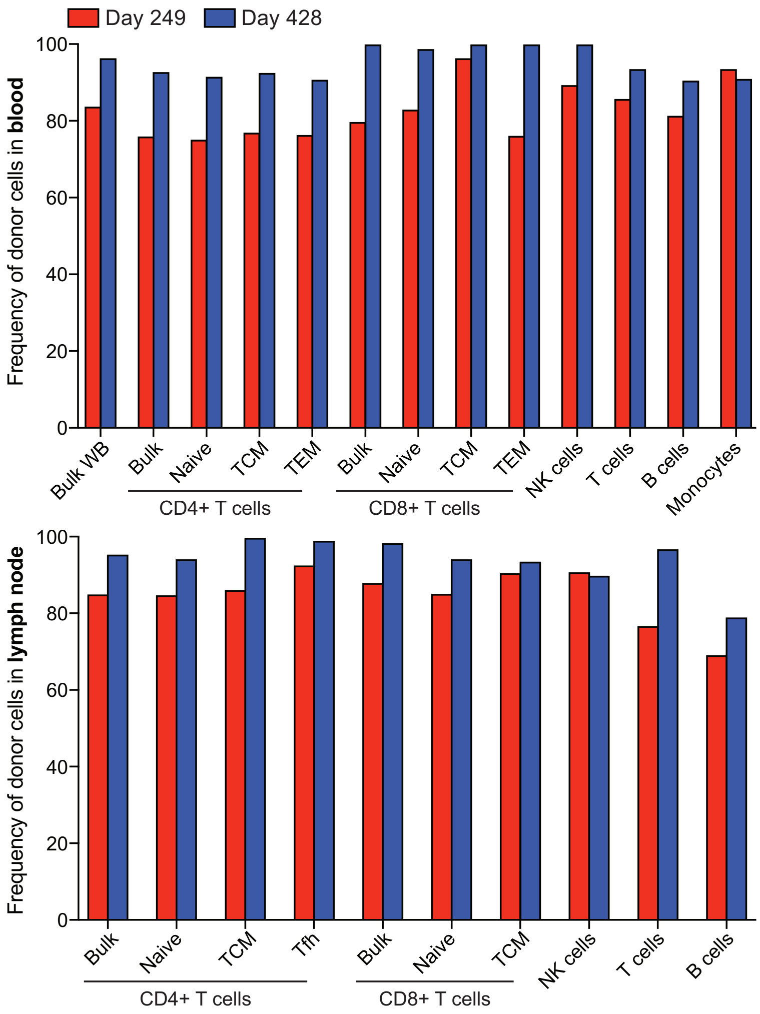

Supplementary figure 12: Longitudinal immune subset chimerism in recipient macaque 33454. Comprehensive immune subset donor chimerism levels in whole blood (**top**) and lymph node (**bottom**) of recipient macaque 33454 at day 249 (red) and day 428 (blue) post-HSCT. Graphs show frequencies of donor-derived cells as measured by Illumina sequencing genomic DNA across SNP 10. Cellular populations were isolated by flow cytometric cell sorting of ACK-treated whole blood prior to genomic DNA extraction. Note donor lymphocyte infusion of $5x10^7$ CD3+ cells/kg was administered at day 380 post-HSCT.

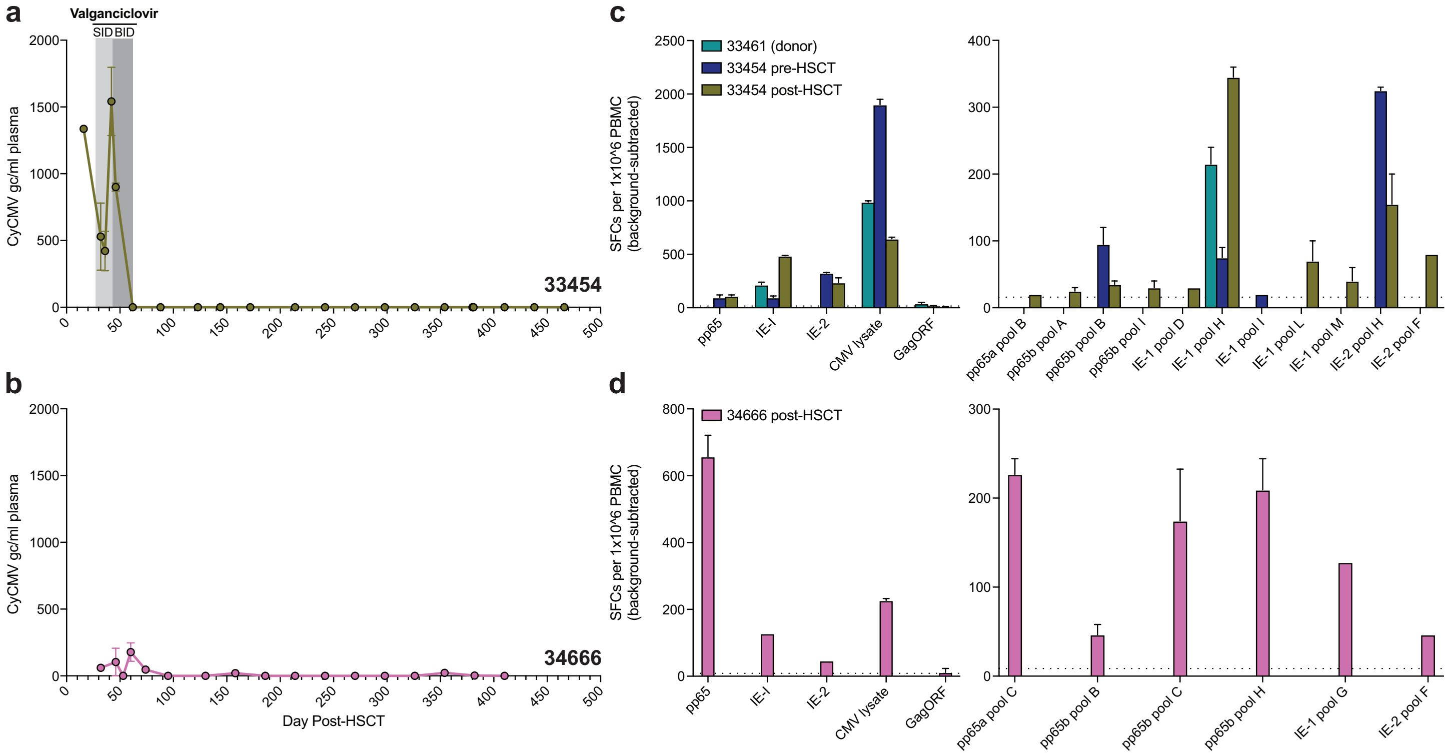

Supplementary figure 13: Cynomolgus macaque CMV (CyCMV) monitoring in recipients with stable engraftment.

(a,b) Longitudinal CyCMV levels in plasma of recipient macaques 33454 **(a)** and 34666 **(b)**, as measured by quantitative PCR detection of viral DNA. Gray boxes indicate SID (once daily, light gray) and BID (twice daily, dark gray) valganciclovir treatment. **(c,d)** IFN-γ ELISPOT detection of CMV-specific T cell responses in PBMC. Responses detected in 33461 (donor for 33454) and 33454 (recipient) pre-HSCT and 466 days post-HSCT are shown in **(c)**. Responses detected in 34666 421 days post-HSCT are shown in **(d).** Graphs display background-subtracted mean±SEM spot-forming cells (SFCs) per million PBMC of two duplicate wells for each antigen. Dotted line indicates the mean response to negative control peptide pool SIVmac239 GagORF for that PBMC sample.

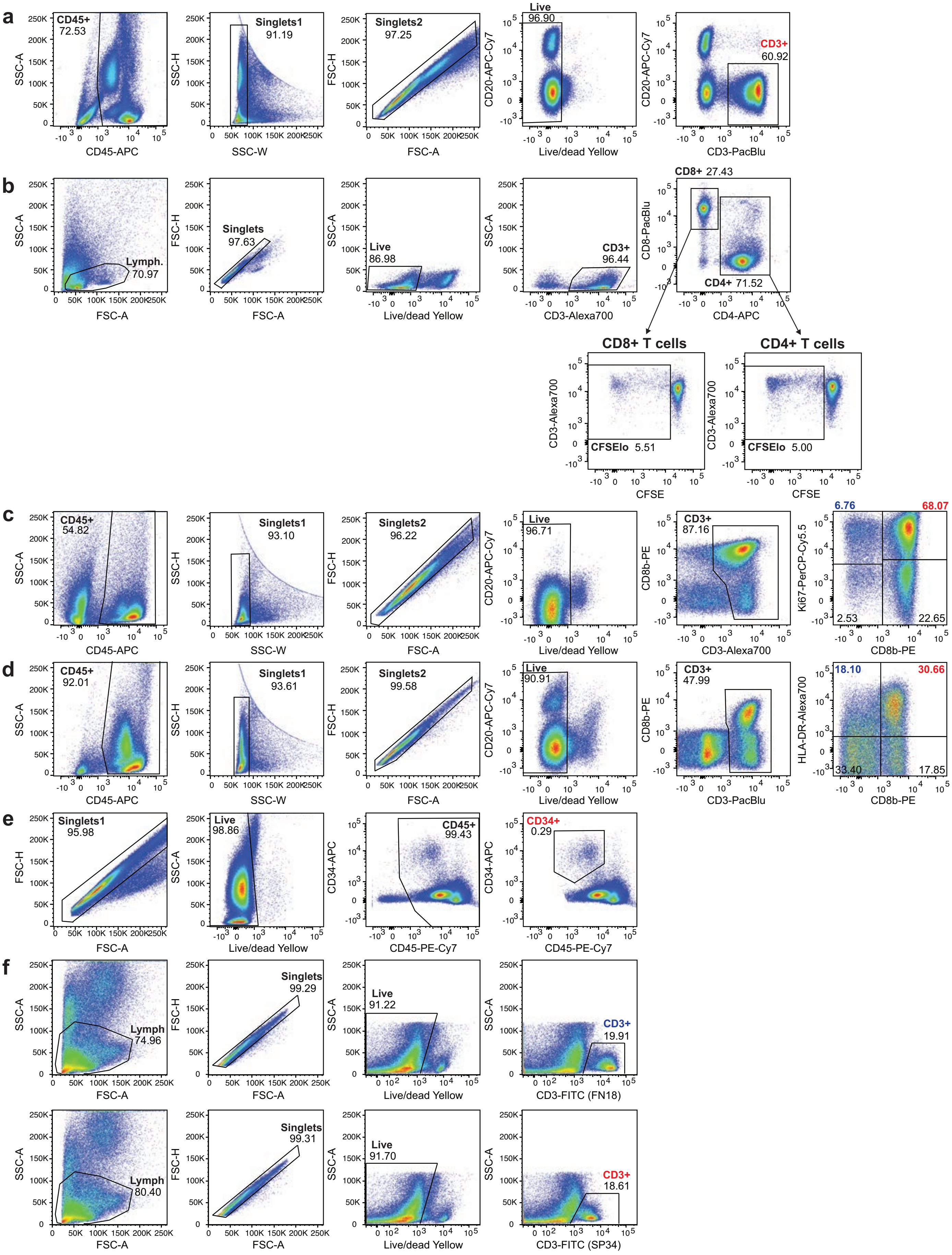
a
CD45+ 72.53
SSC-A
CD45-APC
Singlets1 91.19
SSC-H
SSC-W
Singlets2 97.25
FSC-H
FSC-A
Live 96.90
CD20-APC-Cy7
Live/dead Yellow
CD3+ 60.92
CD20-APC-Cy7
CD3-PacBlu
b
Lymph. 70.97
SSC-A
FSC-A
Singlets 97.63
FSC-H
FSC-A
Live 86.98
SSC-A
Live/dead Yellow
CD3+ 96.44
SSC-A
CD3-Alexa700
CD8+ 27.43
CD4+ 71.52
CD8-PacBlu
CD4-APC
CD8+ T cells
CD4+ T cells
CD3-Alexa700
CFSElo 5.51
CFSE
CD3-Alexa700
CFSElo 5.00
CFSE
c
CD45+ 54.82
SSC-A
CD45-APC
Singlets1 93.10
SSC-H
SSC-W
Singlets2 96.22
FSC-H
FSC-A
Live 96.71
CD20-APC-Cy7
Live/dead Yellow
CD3+ 87.16
CD8b-PE
CD3-Alexa700
6.76
68.07
2.53
22.65
Ki67-PerCP-Cy5.5
CD8b-PE
d
CD45+ 92.01
SSC-A
CD45-APC
Singlets1 93.61
SSC-H
SSC-W
Singlets2 99.58
FSC-H
FSC-A
Live 90.91
CD20-APC-Cy7
Live/dead Yellow
CD3+ 47.99
CD8b-PE
CD3-PacBlu
18.10
30.66
33.40
17.85
HLA-DR-Alexa700
CD8b-PE
e
Singlets1 95.98
FSC-H
FSC-A
Live 98.86
SSC-A
Live/dead Yellow
CD45+ 99.43
CD34-APC
CD45-PE-Cy7
CD34+ 0.29
CD34-APC
CD45-PE-Cy7
f
Lymph 74.96
SSC-A
FSC-A
Singlets 99.29
FSC-H
FSC-A
Live 91.22
SSC-A
Live/dead Yellow
CD3+ 19.91
SSC-A
CD3-FITC (FN18)
Lymph 80.40
SSC-A
FSC-A
Singlets 99.31
FSC-H
FSC-A
Live 91.70
SSC-A
Live/dead Yellow
CD3+ 18.61
SSC-A
CD3-FITC (SP34)

Supplementary figure 14: Gating strategies. Flow cytometry plots show representative gating schemes (gated progressively from left to right) for **(a)** CD3 frequencies determining CD3 counts shown in Figures 1a, 1b, 2a, 3a, **(b)** Mixed lymphocyte reaction T cells shown in Figures 1d, 2h, and 3d, **(c)** Ki67+ T cell frequencies shown in Figure 2g, **(d)** HLA-DR+ T cell frequencies shown in Supplementary Figure 8, **(e)** CD34+ frequencies shown in Supplementary Figure 2, **(f)** CD3 typing histograms shown in Supplementary Figure 4.

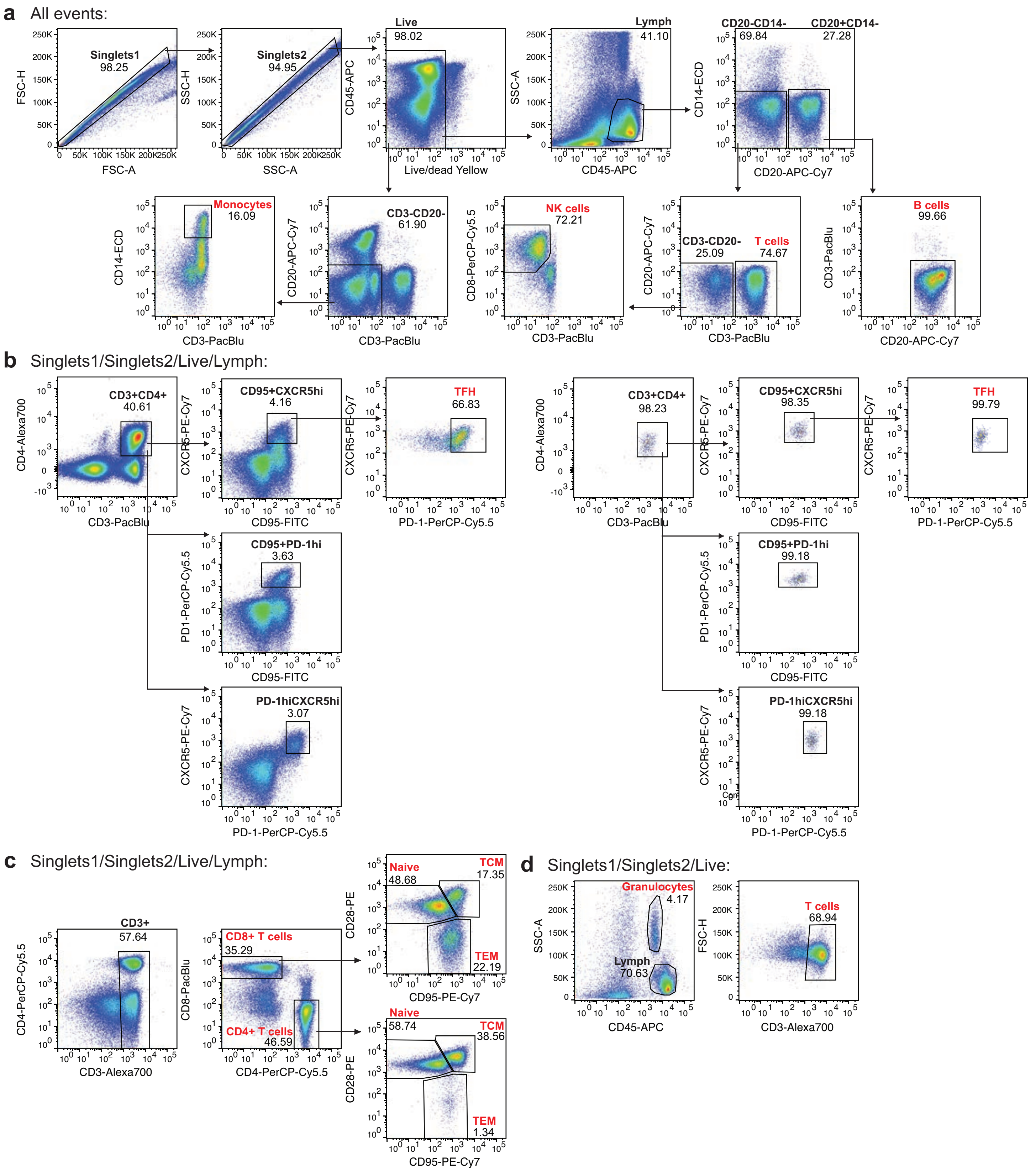

Supplementary figure 15: Gating strategies for sorting. Flow cytometry plots show representative gating schemes for sorting of cellular subsets for chimerism analysis. Plots show sorting strategy for **(a)** T cells, B cells, NK cells, and monocytes as shown in Figure 3c and Supplementary Figure 12, **(b)** T follicular helper cells (post-sort TFH at right) as shown in Figure 3c and Supplementary Figure 12, **(c)** T cell memory subsets as

shown in Figure 3c and Supplementary Figure 12, and **(d)** Granulocytes and T cells as shown in longitudinal chimerism graphs in Figure 3b.

Supplementary table 1: Single nucleotide polymorphisms used for donor chimerism detection assays.

	Animal ID	Allele 1	Allele 2	Context Sequence	Length (bp)	Length after barcoding	F primer	R primer	Chromosome	Start	Stop	Genomic location
**SNP 1**	33450	A	A	CAGGC[A/C]GTGAATCG	213	334	CCAGTCCTTTCTCCAAGTTCTTTTCA	GCACGTTGAGCTGGAGAAGAA	5	16982925	16982985	X16659718.6.D8YO
	33452	C	A									
	33456	C	C									
	34666	C	A									
**SNP 3**	32847	A	A	TGAGACA[A/T]TAGCTTT	216	337	ATTTGATGTATTATCTCCTAGAGAGTCCAAGT	GGGTCCTATGTTAGAAATCAGAATGAAAC	5	53459882	53459942	X50319998.6.D8YO
	32851	T	T									
**SNP 4**	32843	A	G	TATGGGAG[A/G]TCAGCTA	208	329	CCTACAACAGCAGGCTGAACA	TCCTGTTCTACTGGGTCAGACAAT	7	16277084	16277144	X16130672.3.D8YO
	32846	A	A									
	32849	G	G									
	33452	A	A									
	33455	G	G									
**SNP 5**	32843	A	G	GTGCTCTCT[A/G]TAAACC	214	335	GAGAATGAAATTCTTCATCACTGCTTAACC	ACTGAGAGACTGGAGAGATTGAATCA	7	68786667	68786727	X86532773.3.D8YO
	32846	A	A									
	32847	A	G									
	32851	G	G									
	33452	A	G									
	33455	G	G									
**SNP 6**	32843	T	T	TTCAGCA[T/C]AGTTAG	214	335	AGCACTGAGTGATTTTGCTTTGTTT	GGAGTACCTGGGAATGGTTGTG	8	57617203	57617263	X62326623.8.D8YO
	32849	T	C									
	32847	T	T									
	32851	C	C									
	33450	T	C									
	33452	T	T									
**SNP 7**	33452	G	A	CCCAACA[A/G]AAAATCT	214	335	TGAGTGAAAGACAATATTCGGGTGTT	CTTCAGCACCTGGGTATTTTTTAAATATGTT	12	69435595	69435655	X28916342.11.D8Y
	33455	G	G									
	32843	A	G									
	32846	G	G									
**SNP 8**	32843	A	A	AAAGATT[A/C]TCTCCTG	224	345	TCAGGAGGGTAACTTAATATCCATGACA	TCCTTTGCACATAAGCACATATTCAC	12	69435595	69435655	X68169940.11.D8Y
	32849	A	C									
	32847	C	C									
	32851	A	A									
**SNP 10**	33454	C	C	CAGGATC[C/T]CCTCTCTT	221	342	GGGACCTCAAAGGTGATTG	GAAGAGGCAGGCTGTAAAG	12	7112997	7113397	CD43558
	33461	T	C									
	33456	T	C									
	34666	C	C									
