# Supplementary material for: Allogeneic stem cell transplantation in fully MHC-matched Mauritian cynomolgus macaques recapitulates diverse human clinical outcomes
Source: Nat Commun. 2017 Nov 10;8:1418. doi: 10.1038/s41467-017-01631-z (PMC5681693; doi:10.1038/s41467-017-01631-z)
Supplement: Supplementary file 2 — Peer review file [file 41467_2017_1631_MOESM2_ESM.pdf]

Editorial Note: This manuscript has been previously reviewed at another journal that is not operating a transparent peer review scheme. This document only contains reviewer comments and rebuttal letters for versions considered at Nature Communications.
